# Conduction Disturbance, Pacemaker Rates, and Hospital Length of Stay Following Transcatheter Aortic Valve Implantation with the Sapien 3 Valve

**DOI:** 10.1016/j.shj.2022.100019

**Published:** 2022-03-30

**Authors:** Toshiaki Isogai, Shashank Shekhar, Anas M. Saad, Omar M. Abdelfattah, Khaldoun G. Tarakji, Oussama M. Wazni, Ankur Kalra, James J. Yun, Amar Krishnaswamy, Grant W. Reed, Samir R. Kapadia, Rishi Puri

**Affiliations:** Department of Cardiovascular Medicine, Heart, Vascular and Thoracic Institute, Cleveland Clinic, Cleveland, Ohio, USA

**Keywords:** Complete heart block, Length of stay, Permanent pacemaker, Temporary pacemaker, Transcatheter aortic valve implantation

## Abstract

**Background:**

In the absence of randomized data, an expert panel recently proposed an algorithm for conduction disturbance management in transcatheter aortic valve implantation (TAVI) recipients. However, external validations of its recommendations are limited.

**Methods:**

We retrospectively identified 808 patients without a pre-existing pacing device who underwent transfemoral TAVI with the Sapien 3 valve at our institution in 2018-2019. Patients were grouped based on pre-existing conduction disturbance and immediate post-TAVI electrocardiogram. Timing of temporary pacemaker (TPM) removal and hospital discharge were compared with those of the expert panel recommendations to evaluate the associated risk of TPM reinsertion and permanent pacemaker (PPM) implantation.

**Results:**

In most group 1 patients (no electrocardiogram changes without pre-existing right bundle branch block), the timing of TPM removal and discharge were concordant with those of the expert panel recommendations, with low TPM reinsertion (0.8%) and postdischarge PPM (0.8%) rates. In the majority of group 5 patients (procedural high-degree/complete atrioventricular block), TPM was maintained, followed by PPM implantation, compatible with the expert panel recommendations. In contrast, in groups 2-4 (pre-existing/new conduction disturbances), earlier TPM removal than recommended by the expert panel (mostly, immediately after procedure) was feasible in 97.5%-100% of patients, with a low TPM reinsertion rate (0.0%-1.8%); earlier discharge was also feasible in 50.0%-65.5%, with a low 30-day postdischarge PPM rate (0.0%-2.8%) and no 30-day death.

**Conclusions:**

Early TPM removal and discharge after TAVI appear safe and feasible in the majority of cases. These data may provide a framework for an early, streamlined hospital discharge plan for TAVI recipients, optimizing both cost savings and patient safety.

## Introduction

Despite advances in transcatheter heart valve technologies and improved implantation techniques, a standardized approach to managing conduction system diseases during transcatheter aortic valve implantation (TAVI) has thus far remained elusive, lacking prospective randomized clinical evidence. Considerable variations in local practice, ranging from valve choice, periprocedural management of temporary pacemaker (TPM), and thresholds for permanent pacemaker (PPM) implantation, impact not only PPM rates after TAVI but also hospital length of stay.^1^ Furthermore, PPM rates during the period between hospital discharge and 30 days after TAVI have increased in recent times in the context of shorter hospital stays.^2^^,^^3^

In response to the lack of uniformity in clinical practice for managing conduction system disturbances in TAVI recipients, in 2019, a scientific expert panel was independently convened to propose a detailed algorithm strategy for managing conduction disturbances in TAVI recipients. These recommendations pertained to the timing of TPM removal and hospital discharge upon the basis of baseline and periprocedural electrocardiogram (ECG) changes during TAVI.^4^ While the document served as an important sentinel step for standardizing clinical practice, its content was based largely upon expert opinion owing to the lack of high-quality contemporary clinical experience. A recent external validation of the expert panel recommendations at a single Swiss center was limited by their prolonged length of hospital stay as well as their lack of data on TPM removal timing^5^; questionable relevance to contemporary US TAVI practice and length of stay metrics.^6^ The present analysis categorizes the conduction system disease, clinical workflow, and outcomes of our contemporary TAVI recipients according to the proposed criteria set forth by the expert panel. Our timing of TPM removal (and reinsertion if required), in-hospital and 30-day PPM rates, and hospital length of stay were analyzed and systematically compared against the proposed expert panel recommendations.

## Methods

### Study Design and Data Collection

The present study is a retrospective analysis conducted at the Cleveland Clinic. Detailed data on baseline patient characteristics, ECG, procedural characteristics, and in-hospital/postdischarge events were extracted from our prospectively collected institutional registries or were manually extracted from electronic medical records. Outcomes were defined according to the Valve Academic Research Consortium-2 criteria.^7^ The study was approved by the institutional review board of the Cleveland Clinic, and the requirement of informed consent was waived due to the retrospective nature of this study.

### Periprocedural Workflow

Since April 2017, we have systematically introduced a high valve deployment technique to decrease the post-TAVI PPM risk (Supplemental Figure 1).^8^ Typically, the TPM is removed immediately following the TAVI procedure regardless of pre-existing or new conduction disturbances unless persistent complete heart block (CHB) exists or maintaining TPM is judged as necessary by the operating physician. Upon leaving the hybrid operating room, patients are transferred to a step-down unit (or intensive care unit if necessary) and are continuously monitored on telemetry. If new or worsening conduction disturbance or bradyarrhythmia is observed, TPM reinsertion is typically considered, and our electrophysiology team is rapidly consulted for the need of PPM implantation. At our institution, all patients who underwent TAVI before August 2018 remained in hospital at least until post-TAVI day 2 as part of routine practice, whereas since August 2018, patients began to be discharged routinely on day 1 after TAVI if judged possible by our heart team. Telemetry is continued until discharge. An ECG-patch (Zio Patch; iRhythm, San Francisco, California) is used for ambulatory ECG monitoring following discharge at the discretion of operating physicians or as part of our prior prospective study (Brady-TAVR, NCT03180073). Following hospital discharge, outpatient follow-up is typically arranged within 3-7 days.

### Patient Selection and Grouping

**Figure 1:**
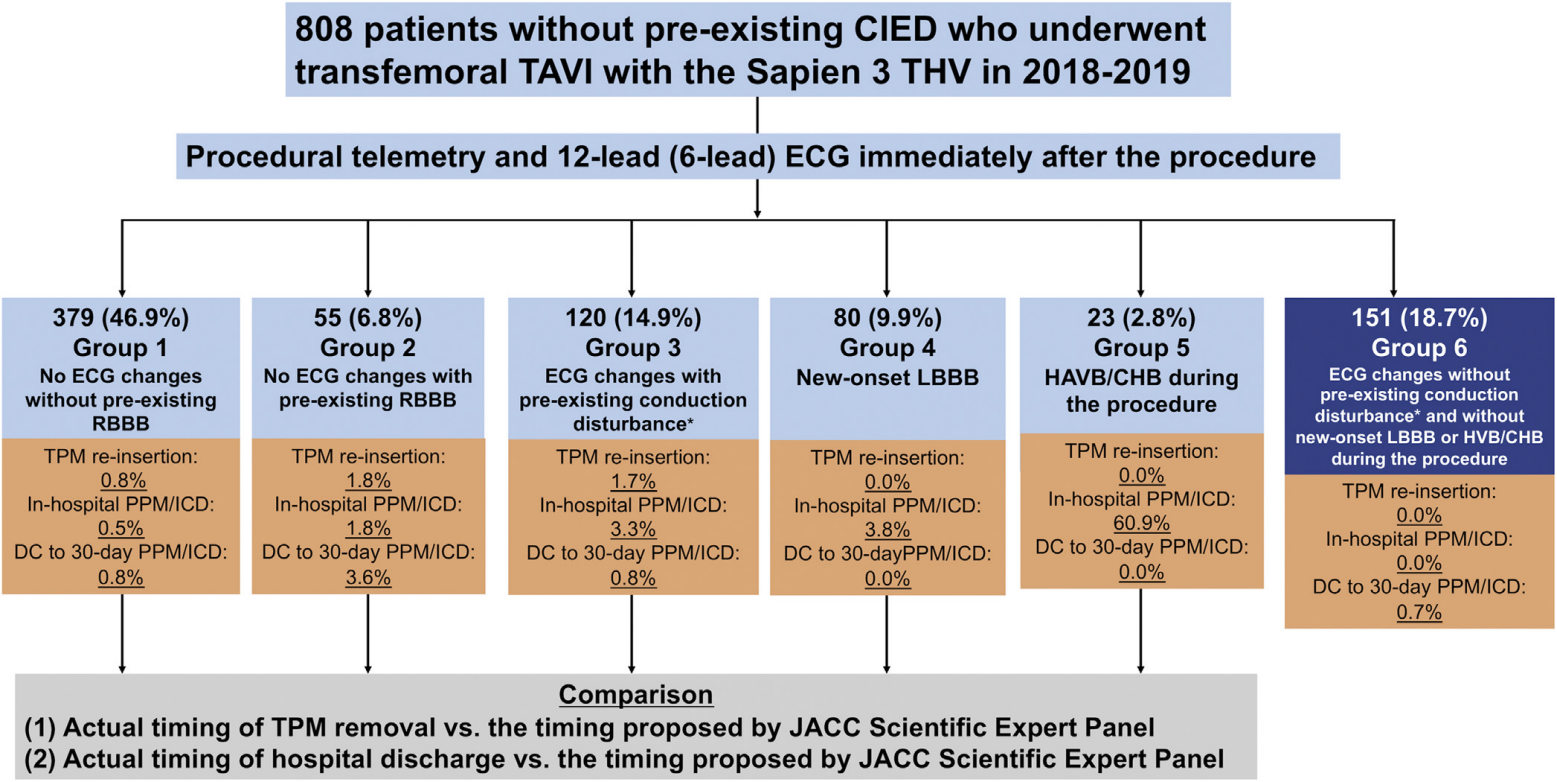
**Grouping of patients according to the JACC expert panel recommendations.** ∗Conduction disturbance includes RBBB, LBBB, interventricular conduction delay, and first-degree AVB. Abbreviations: AVB, atrioventricular block; CHB, complete heart block; CIED, cardiac implantable electronic device; DC, discharge; ECG, electrocardiogram; HAVB, high-degree atrioventricular block; ICD, implantable cardioverter defibrillator; JACC, Journal of the American College of Cardiology; LBBB, left bundle branch block; PPM, permanent pacemaker; RBBB, right bundle branch block; TAVI, transcatheter aortic valve implantation; THV, transcatheter heart valve; TPM, temporary pacemaker.

The present analysis included consecutive patients aged ≥18 years who underwent TAVI at our institution between January 2018 and December 2019. No patient underwent pre-TAVI “prophylactic” PPM implantation as part of their pre-TAVI evaluation. We excluded patients who underwent nontransfemoral TAVI; those who were admitted for other treatments/examinations before TAVI and thereafter underwent TAVI during the same hospitalization (i.e., inpatient TAVI); those with pre-existing cardiac implantable electronic devices; and those without identifiable 12-lead (or 6-lead) ECG immediately after the TAVI procedure or at day 1 after TAVI (these ECGs are necessary for grouping and determining the proposed timing according to the expert panel algorithm).^4^ All eligible patients underwent transfemoral TAVI on the day of admission (i.e., outpatient TAVI). We also excluded patients who underwent TAVI with a self-expanding valve because of the small sample size during this study period (n = 37). Eligible patients were transfemoral TAVI recipients with the Sapien 3 transcatheter heart valve (Edwards Lifesciences, Irvine, California) and were grouped according to the expert panel algorithm.^4^ Meanwhile, none of the 5 groups defined by the algorithm included patients without pre-existing right bundle branch block (RBBB), left bundle branch block (LBBB), or nonspecific interventricular conduction delay with QRS duration ≥120 ​ms or first-degree atrioventricular block who developed ECG changes (defined as an increase in PR or QRS segment duration ≥20 ​ms) but not new-onset LBBB or high-degree atrioventricular block (HAVB)/CHB during the procedure. Therefore, such patients were newly defined as group 6 (Figure 1).

### ECG Assessment

Twelve-lead (or 6-lead) ECGs were undertaken, at minimum, at 3 different time points (baseline before TAVI, immediately after TAVI, and on day 1 after TAVI), and telemetry data were examined in all eligible patients. All ECG findings were evaluated according to the standard definitions and guidelines by the American Heart Association, American College of Cardiology Foundation, and Heart Rhythm Society recommendations.^9^^,^^10^ According to the expert panel definitions, procedural HAVB/CHB was defined as any HAVB/CHB episode occurring during the TAVI procedure; delayed HAVB/CHB was defined as any HAVB/CHB episode occurring after the patient had left the procedure room and within 30 days after the procedure.

### Actual vs. Proposed Timing of TPM Removal and Hospital Discharge After TAVI

We compared the actual timing of TPM removal with the timing proposed by the expert panel in each of groups 1-5.^4^ If the actual timing was the same as that recommended by the expert panel, the TPM removal was defined as “concordant TPM removal.” If the actual timing was earlier or later than recommended, the TPM removal was defined as “early TPM removal” and “delayed TPM removal,” respectively. If TPM was maintained until the implantation of PPM or implantable cardioverter defibrillator (ICD), the patients were categorized as “TPM leading directly to PPM/ICD.” We compared the rates of TPM reinsertion and in-hospital PPM/ICD implantation among the early, concordant, and delayed TPM removal groups. In a similar manner, we compared the actual timing of hospital discharge with the timing recommended by the expert panel^4^ and categorized the patients as “early discharge,” “concordant discharge,” and “delayed discharge.” If a PPM or ICD was implanted during index hospitalization, the patients were categorized as “discharge after PPM/ICD.” We compared the rates of postdischarge PPM/ICD implantation and all-cause death among the early, concordant, and delayed discharge groups. Postdischarge outcomes were collected through our institutional electronic medical records in Epic (Epic Systems Corporation) as well as outside hospital records using the “Care Everywhere” function in Epic.

### Statistical Analysis

Categorical variables were compared using chi-square test or Fisher exact test. Continuous variables were compared using the Mann-Whitney *U* test or Student *t*-test for 2-group comparison and Kruskal-Wallis test or analysis of variance for ≥3-group comparison as appropriate. A 2-sided *p* value of <0.05 was set as significant in all hypothesis tests. All statistical analyses were conducted using IBM SPSS Statistics, version 26 (IBM Corp, Armonk, New York) and Stata 15.0 (StataCorp, College Station, Texas).

## Results

### Baseline Characteristics and In-Hospital Adverse Events

A total of 808 eligible transfemoral TAVI recipients (mean age 78.8 ± 9.4 years; males 59.4%) were identified (Supplemental Figure 2) and categorized into 6 groups (Figure 1). Sex distribution differed significantly across the groups, but there were no significant differences in age and surgical risk score (Table 1). Baseline ECG findings differed significantly across the groups as per the expert panel algorithm. A larger valve was more often used in group 5. In-hospital adverse events did not differ significantly across the groups. No patient died during index hospitalization or within 30 days after TAVI. The median length of stay was 1 day (interquartile range: 1, 2 days), 54.8% of the patients were discharged on day 1, and 95% of the patients were discharged home (Supplemental Table 1).

**Table 1:** Patient and procedural characteristics

	Groups 1-6	Group 1 (no ECG changes without pre-existing RBBB)	Group 2 (no ECG changes with pre-existing RBBB)	Group 3 (ECG changes with pre-existing conduction disturbance∗)	Group 4 (new-onset LBBB)	Group 5 (HAVB/CHB during the procedure)	Group 6 (ECG changes without pre-existing conduction disturbance∗ and without new-onset LBBB or HVB/CHB)	*p* value
	(N = 808)	(n = 379)	(n = 55)	(n = 120)	(n = 80)	(n = 23)	(n = 151)	
Patient characteristics								
Age, y	78.8 ± 9.4	78.1 ± 9.8	78.7 ± 8.4	80.5 ± 8.8	79.9 ± 9.9	78.6 ± 9.0	78.5 ± 9.2	0.20
Male	480 (59.4)	225 (59.4)	42 (76.4)	84 (70.0)	42 (52.5)	17 (73.9)	70 (46.4)	<0.001
Body mass index, kg/m^2^	29.0 ± 6.5	28.8 ± 6.6	29.1 ± 6.9	29.7 ± 6.7	29.4 ± 6.5	29.3 ± 5.9	28.9 ± 6.0	0.83
STS risk score	4.37	4.20	3.70	4.51	5.05	5.50	4.04	0.19
	(2.86, 6.73)	(2.82, 6.69)	(2.90, 7.00)	(3.10, 6.91)	(3.42, 6.70)	(3.84, 7.73)	(2.80, 6.17)	
History of syncope	21 (2.6)	8 (2.1)	0 (0.0)	4 (3.3)	4 (5.0)	1 (4.3)	4 (2.6)	0.41
History of atrial fibrillation/flutter	285 (35.3)	156 (41.2)	21 (38.2)	29 (24.2)	36 (45)	12 (52.2)	31 (20.5)	<0.001
Prior PCI	262 (32.4)	114 (30.1)	24 (43.6)	35 (29.2)	29 (36.3)	9 (39.1)	51 (33.8)	0.32
Prior CABG	143 (17.7)	63 (16.6)	14 (25.5)	31 (25.8)	14 (17.5)	2 (8.7)	19 (12.6)	0.040
Prior myocardial infarction	142 (17.6)	63 (16.6)	11 (20.0)	19 (15.8)	18 (22.5)	6 (26.1)	25 (16.6)	0.61
Prior stroke	113 (14.0)	50 (13.2)	8 (14.5)	18 (15.0)	13 (16.3)	6 (26.1)	18 (11.9)	0.53
Prior peripheral arterial disease	554 (68.6)	253 (66.8)	42 (76.4)	87 (72.5)	52 (65.0)	20 (87.0)	100 (66.2)	0.19
ESRD on dialysis	22 (2.7)	12 (3.2)	0 (0.0)	2 (1.7)	1 (1.3)	1 (4.3)	6 (4.0)	0.55
Hypertension	716 (88.6)	335 (88.4)	54 (98.2)	107 (89.2)	69 (86.3)	20 (87.0)	131 (86.8)	0.19
Diabetes mellitus	279 (34.5)	127 (33.5)	17 (30.9)	44 (36.7)	33 (41.3)	9 (39.1)	49 (32.5)	0.72
NYHA functional class III or IV	542 (67.1)	255 (67.3)	36 (65.5)	83 (69.2)	59 (73.8)	14 (60.9)	95 (62.9)	0.61
Baseline rhythm: atrial fibrillation	104 (12.9)	82 (21.6)	6 (10.9)	2 (1.7)	5 (6.3)	3 (13.0)	6 (4.0)	<0.001
Baseline PR interval, ms (N = 681)†	176 (158, 202)	174 (154, 200)	184 (161, 204)	208 (184, 236)	184 (161, 202)	186 (172, 208)	164 (150, 179)	<0.001
Baseline QRS duration, ms	98 (88, 118)	96 (88, 108)	144 (134, 156)	124 (97, 146)	100 (89, 111)	138 (118, 149)	90 (84, 96)	<0.001
Pre-existing first-degree AVB	181 (22.4)	65 (17.2)	15 (27.3)	78 (65.0)	18 (22.5)	5 (21.7)	0 (0.0)	<0.001
Pre-existing RBBB	115 (14.2)	0 (0.0)	55 (100.0)	43 (35.8)	1 (1.3)	16 (69.6)	0 (0.0)	<0.001
LVEF, %	58.6 ± 10.2	58.5 ± 9.9	58.7 ± 10.3	57.3 ± 11.8	57.8 ± 10.9	54.7 ± 13.2	60.7 ± 8.6	0.031
Aortic valve mean gradient, mmHg	42.1 ± 13.9	41.6 ± 13.7	40.8 ± 14.6	44.0 ± 14.9	41.2 ± 14.7	42.8 ± 11.7	42.6 ± 13.4	0.59
Aortic valve area, cm^2^ (N = 770)	0.74 ± 0.18	0.73 ± 0.20	0.76 ± 0.17	0.72 ± 0.16	0.71 ± 0.17	0.71 ± 0.15	0.77 ± 0.18	0.16
Bicuspid aortic valve	54 (6.7)	30 (7.9)	3 (5.5)	1 (0.8)	3 (3.8)	5 (21.7)	12 (7.9)	0.003
Failed bioprosthetic valve	54 (6.7)	25 (6.6)	6 (10.9)	10 (8.3)	1 (1.3)	0 (0.0)	12 (7.9)	0.13
Procedural characteristics								
Nonelective procedure‡	28 (3.5)	15 (4.0)	0 (0.0)	2 (1.7)	4 (5.0)	2 (8.7)	5 (3.3)	0.27
Anesthesia type								0.27
Conscious sedation	803 (99.4)	378 (99.7)	55 (100.0)	119 (99.2)	80 (100.0)	23 (100.0)	148 (98)	
General anesthesia	5 (0.6)	1 (0.3)	0 (0.0)	1 (0.8)	0 (0.0)	0 (0.0)	3 (2.0)	
Valve size								<0.001
20 mm	35 (4.3)	13 (3.4)	3 (5.5)	3 (2.5)	5 (6.3)	0 (0.0)	11 (7.3)	
23 mm	301 (37.3)	146 (38.5)	12 (21.8)	38 (31.7)	30 (37.5)	5 (21.7)	70 (46.4)	
26 mm	290 (35.9)	141 (37.2)	29 (52.7)	46 (38.3)	20 (25.0)	6 (26.1)	48 (31.8)	
29 mm	182 (22.5)	79 (20.8)	11 (20.0)	33 (27.5)	25 (31.3)	12 (52.2)	22 (14.6)	
Predilation	77 (9.5)	39 (10.3)	3 (5.5)	14 (11.7)	10 (12.5)	4 (17.4)	7 (4.6)	0.089
Postdilation	365 (45.2)	170 (44.9)	26 (47.3)	60 (50.0)	37 (46.3)	10 (43.5)	62 (41.1)	0.80
Cerebral embolic protection	779 (96.4)	367 (96.8)	53 (96.4)	118 (98.3)	73 (91.3)	20 (87.0)	148 (98.0)	0.020

*Notes*. Values are n (%), median (quartile 1, quartile 3), or mean ± standard deviation.

Abbreviations: AVB, atrioventricular block; CABG, coronary artery bypass grafting; CHB, complete heart block; ECG, electrocardiogram; ESRD, end-stage renal disease; HAVB, high-degree atrioventricular block; LBBB, left bundle branch block; LVEF, left ventricular ejection fraction; NYHA, New York Heart Association; PCI, percutaneous coronary intervention; RBBB, right bundle branch block; STS, Society of Thoracic Surgeons.

∗ Conduction disturbance includes RBBB, LBBB, interventricular conduction delay, or/and first-degree AVB.

† Includes patients with sinus rhythm.

‡ Defined as the procedure performed earlier than initially planned in a patient who was admitted with worsening symptoms or/and cardiac function.

### Overall TPM Reinsertion, HAVB/CHB, and PPM/ICD Implantation

Overall, TPM was removed at the end of TAVI in 96.9% of the patients (Supplemental Table 2). During hospitalization, HAVB/CHB occurred in 34 (4.2%) patients, including 23 procedural HAVB/CHBs and 11 delayed HAVB/CHBs (same day of TAVI n = 6; day 1 n = 1; day 2 n = 1; day 3 n = 2; day 4 n = 1). TPM was reinserted in 0.7% (n = 6: all for delayed HAVB/CHB). During hospitalization, PPM/ICD was implanted in 24 (3.0%) patients (21 for HAVB/CHB and 3 for sick sinus syndrome). Between discharge and 30 days after TAVI, there were 2 (0.2%) delayed HAVB/CHB events (on day 4 and day 16) and 7 (0.9%) PPM/ICD implantations (Supplemental Table 3). Overall, the 30-day HAVB/CHB rate was 4.3% (procedural 2.8%, delayed 1.5%). The overall 30-day PPM/ICD rate was 3.7%.

### Timing of TPM Removal and Hospital Discharge According to Each Group

Granular data on the timing of TPM removal and hospital discharge are shown in Supplemental Figures 3 and 4.

#### Group 1 (No ECG Changes Without Pre-Existing RBBB)

**Figure 2:**
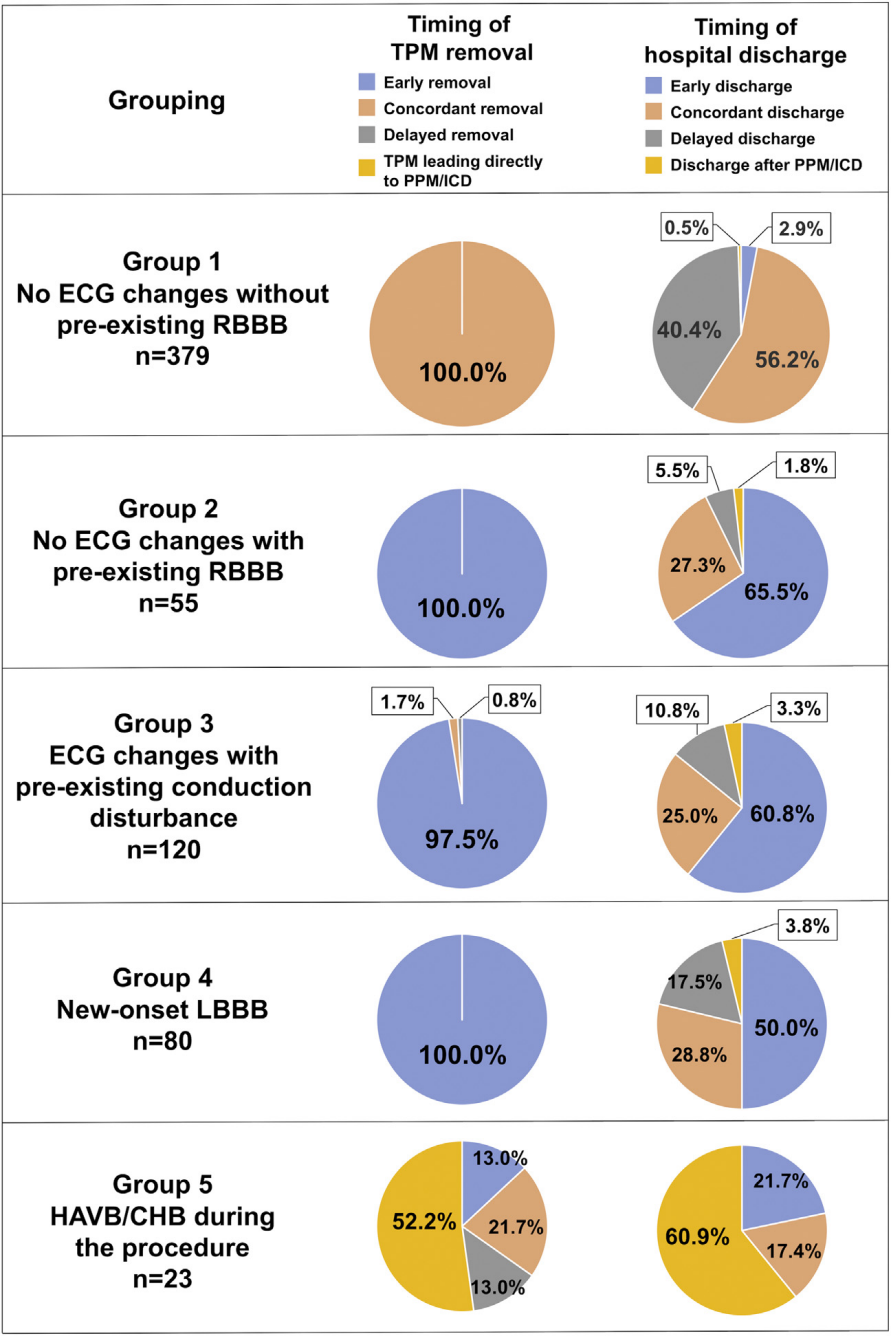
**Actual timing vs. expert panel proposed timing of TPM removal and hospital discharge after TAVI in group 1-5.** Abbreviations: CHB, complete heart block; ECG, electrocardiogram; HAVB, high-degree atrioventricular block; ICD, implantable cardioverter defibrillator; LBBB, left bundle branch block; PPM, permanent pacemaker; RBBB, right bundle branch block; TAVI, transcatheter aortic valve implantation; TPM, temporary pacemaker.

TPM was removed at the end of TAVI in all patients, concordant with the expert panel recommendation in all patients. Concordant hospital discharge was observed in 56.2% of the patients (Figure 2). TPM was reinserted in 3 (0.5%) patients, none of whom received PPM/ICD implantation due to a lack of recurrent HAVB/CHB. Two patients received PPM/ICD implantation (both dual-chamber PPM for sick sinus syndrome) during the index hospitalization (Table 2). Three patients received PPM/ICD implantation within 30 days after delayed discharge (Table 3).

**Table 2:** Incidence of TPM reinsertion and PPM/ICD implantation according to timing of TPM removal relative to expert panel recommendations

	Overall	Actual timing vs. expert panel proposed timing of TPM removal			*p* value
		Early removal	Concordant removal	Delayed removal	
Group 1 (no ECG changes without pre-existing RBBB)					
No. of patients	379	0	379	0	
TPM reinsertion	3 (0.8)	–	3 (0.8)	–	–
Resulting in PPM/ICD implantation	0 (0.0)	–	0 (0.0)	–	–
No TPM reinsertion, but requiring PPM/ICD implantation	2 (0.5)	–	2 (0.5)	–	–
In-hospital PPM/ICD implantation, total	2 (0.5)	–	2 (0.5)	–	–
Group 2 (no ECG changes with pre-existing RBBB)					
No. of patients	55	55	0	0	
TPM reinsertion	1 (1.8)	1 (1.8)	–	–	–
Resulting in PPM/ICD implantation	1 (1.8)	1 (1.8)	–	–	–
No TPM reinsertion, but requiring PPM/ICD implantation	0 (0.0)	0 (0.0)	–	–	–
In-hospital PPM/ICD implantation, total	1 (1.8)	1 (1.8)	–	–	–
Group 3 (ECG changes with pre-existing conduction disturbance)					
No. of patients	120	117	2	1	
TPM reinsertion	2 (1.7)	2 (1.7)	0 (0.0)	0 (0.0)	1.00
Resulting in PPM/ICD implantation	2 (1.7)	2 (1.7)	0 (0.0)	0 (0.0)	1.00
No TPM reinsertion, but requiring PPM/ICD implantation	2 (1.7)	2 (1.7)	0 (0.0)	0 (0.0)	1.00
In-hospital PPM/ICD implantation, total	4 (3.3)	4 (3.4)	0 (0.0)	0 (0.0)	1.00
Group 4 (new-onset LBBB)					
No. of patients	80	80	0	0	
TPM reinsertion	0 (0.0)	0 (0.0)	–	–	–
Resulting in PPM/ICD implantation	–	–	–	–	–
No TPM reinsertion, but requiring PPM/ICD implantation	3 (3.8)	3 (3.8)	–	–	–
In-hospital PPM/ICD implantation, total	3 (3.6)	3 (3.6)	–	–	–
Group 5 (HAVB/CHB during the procedure)					
No. of patients	11	3	5	3	–
TPM reinsertion	0 (0.0)	0 (0.0)	0 (0.0)	0 (0.0)	–
Resulting in PPM/ICD implantation	–	–	–	–	–
No TPM reinsertion, but requiring PPM/ICD implantation	2 (18.2)	2 (66.7)	0 (0.0)	0 (0.0)	0.11
In-hospital PPM/ICD implantation, total	2 (18.2)	2 (66.7)	0 (0.0)	0 (0.0)	0.11

*Notes*. Values are n (%). Patients in group 6 (n = 151) and patients who received PPM/ICD implantation directly after maintaining TPM (n = 12) were not included in this table.

Abbreviations: CHB, complete heart block; ECG, electrocardiogram; HAVB, high-degree atrioventricular block; ICD, implantable cardioverter defibrillator; PPM, permanent pacemaker; RBBB, right bundle branch block; TPM, temporary pacemaker.

**Table 3:** Incidence of postdischarge PPM/ICD implantation and all-cause death according to timing of hospital discharge relative to expert panel recommendations

	Overall	Actual timing vs. expert panel proposed timing of hospital discharge			*p* value
		Early discharge	Concordant discharge	Delayed discharge	
Group 1 (no ECG changes without pre-existing RBBB)					
No. of patients	377	11	213	153	
Discharge to 30-d PPM/ICD implantation	3 (0.8)	0 (0.0)	0 (0.0)	3 (2.0)	0.15
Discharge to 30-d death	0 (0.0)	0 (0.0)	0 (0.0)	0 (0.0)	–
31 to 180-d PPM/ICD implantation	2 (0.5)	0 (0.0)	2 (0.9)	0 (0.0)	0.54
31 to 180-d Death	7 (1.9)	0 (0.0)	3 (1.4)	4 (2.6)	0.56
Group 2 (no ECG changes with pre-existing RBBB)					
No. of patients	54	36	15	3	
Discharge to 30-d PPM/ICD implantation	2 (3.7)	1 (2.8)	1 (6.7)	0 (0.0)	0.56
Discharge to 30-d death	0 (0.0)	0 (0.0)	0 (0.0)	0 (0.0)	–
31 to 180-d PPM/ICD implantation	0 (0.0)	0 (0.0)	0 (0.0)	0 (0.0)	–
31 to 180-d Death	0 (0.0)	0 (0.0)	0 (0.0)	0 (0.0)	–
Group 3 (ECG changes with pre-existing conduction disturbance)					
No. of patients	116	73	30	13	
Discharge to 30-d PPM/ICD implantation	1 (0.9)	1 (1.4)	0 (0.0)	0 (0.0)	1.00
Discharge to 30-d death	0 (0.0)	0 (0.0)	0 (0.0)	0 (0.0)	–
31 to 180-d PPM/ICD implantation	4 (3.4)	1 (1.4)	1 (3.3)	2 (15.4)	0.044
31 to 180-d Death	3 (2.6)	1 (1.4)	2 (6.7)	0 (0.0)	0.31
Group 4 (new-onset LBBB)					
No. of patients	77	40	23	14	
Discharge to 30-d PPM/ICD implantation	0 (0.0)	0 (0.0)	0 (0.0)	0 (0.0)	–
Discharge to 30-d death	0 (0.0)	0 (0.0)	0 (0.0)	0 (0.0)	–
31 to 180-d PPM/ICD implantation	1 (1.3)	1 (2.5)	0 (0.0)	0 (0.0)	1.00
31 to 180-d Death	2 (2.6)	1 (2.5)	0 (0.0)	1 (7.1)	0.42
Group 5 (HAVB/CHB during the procedure)					
No. of patients	9	5	4	0	–
Discharge to 30-d PPM/ICD implantation	0 (0.0)	0 (0.0)	0 (0.0)	0 (0.0)	–
Discharge to 30-d death	0 (0.0)	0 (0.0)	0 (0.0)	0 (0.0)	–
31 to 180-d PPM/ICD implantation	0 (0.0)	0 (0.0)	0 (0.0)	0 (0.0)	–
31 to 180-d Death	0 (0.0)	0 (0.0)	0 (0.0)	0 (0.0)	–

*Notes*. Values are n (%). Patients in group 6 (n = 151) and patients who received PPM/ICD implantation during index hospitalization (n = 24) were not included in this table. Discharge to 30-d events includes events between discharge and 30 days following transcatheter aortic valve implantation. Thirty one to 180-d events includes events between 31 days and 180 days following transcatheter aortic valve implantation.

Abbreviations: CHB, complete heart block; ECG, electrocardiogram, HAVB; high-degree atrioventricular block, ICD; implantable cardioverter defibrillator, LBBB; left bundle branch block, PPM; permanent pacemaker; RBBB, right bundle branch block.

#### Group 2 (No ECG Changes With Pre-Existing RBBB)

TPM was removed at the end of TAVI in all patients, discordant with the expert panel recommendations (which suggested next-day or later removal). Early hospital discharge relative to the expert panel recommendation was observed in 65.5% of the patients (Figure 2). TPM was reinserted in 1 (1.8%) patient due to delayed CHB, resulting in PPM implantation (Table 2). In this group, only this patient received PPM/ICD implantation during the index hospitalization. Two patients required PPM implantation within 30 days after discharge (1 after early discharge and 1 after concordant timing of discharge) (Table 3).

#### Group 3 (ECG Changes With Pre-Existing Conduction Disturbance)

TPM was removed at the end of TAVI in 96.7% of patients. As a result, 97.5% of patients had early (discordant) TPM removal relative to the expert panel recommendations. Early (discordant) hospital discharge relative to the expert panel recommendations was observed in 60.8% of patients (Figure 2). TPM was reinserted in 2 (1.7%) patients due to HAVB/CHB after early TPM removal, both resulting in PPM implantation. An additional 2 patients received PPM implantation (1 for delayed HAVB/CHB and 1 for sick sinus syndrome) without receiving TPM reinsertion after early TPM removal (Table 2). In total, 4 patients received PPM/ICD implantation (all, dual-chamber PPM) during the index hospitalization. One patient required PPM implantation within 30 days after early discharge (Table 3).

#### Group 4 (New-Onset LBBB)

TPM was removed at the end of TAVI in all patients, discordant (early) to the expert panel recommendation (next-day or later removal). Early discharge compared with the expert panel recommendation was observed in half of the patients (Figure 2). No TPM was reinserted. Meanwhile, a total of 3 patients received PPM/ICD implantation (all, dual-chamber PPM for delayed HAVB/CHB) without receiving TPM reinsertion during the index hospitalization (Table 2). No patient required PPM implantation within 30 days after discharge (Table 3).

#### Group 5 (HAVB/CHB During the Procedure)

In this particular group, 12 (52.2%) out of 23 patients received PPM/ICD implantation directly after maintaining the TPM. In total, 14 (60.9%) patients received PPM/ICD implantation during the index hospitalization and were discharged thereafter (Figure 2). Nine patients were discharged without PPM/ICD implantation due to a lack of recurrent HAVB/CHB and did not require PPM/ICD implantation after discharge (Tables 2 and 3).

Reasons for delayed discharge relative to expert panel recommendations are shown in Supplemental Table 4. Overall, the most frequent reason for delayed discharge was our practice of routine observation until day 2 in group 1 patients before August 2018 (26.3%). Conduction disturbance or bradycardia was observed as a reason for delayed discharge in 12.0%.

### Feasibility and Safety of TPM Removal at the End of TAVI and Next-Day Discharge

Except for group 5, TPM was removed at the end of TAVI procedure in most patients (97%-100%) with a low rate of TPM reinsertion (0.0%-1.8%). Also, apart from group 5, the majority of patients (40%-66%) were discharged on post-TAVI day 1 with a low rate of discharge-to-30-day PPM/ICD implantation (0.0-2.9%) and no death (Supplemental Table 5).

### Preprocedural and Postprocedural Conduction Disturbances and Delayed HAVB/CHB

Patients with pre-existing RBBB had a high overall 30-day HAVB/CHB rate (15.7% [18/115]; Supplemental Table 6), but 88.9% (16/18) of the HAVB/CHB episodes occurred during the TAVI procedure. Pre-existing RBBB had a low sensitivity (17%) in predicting delayed HAVB/CHB; other pre-existing or new conduction disturbances also had relatively low sensitivity (0-54%) in predicting delayed HAVB/CHB (Supplemental Table 7).

### Recategorized Groups and Modified Algorithm

**Figure 3:**
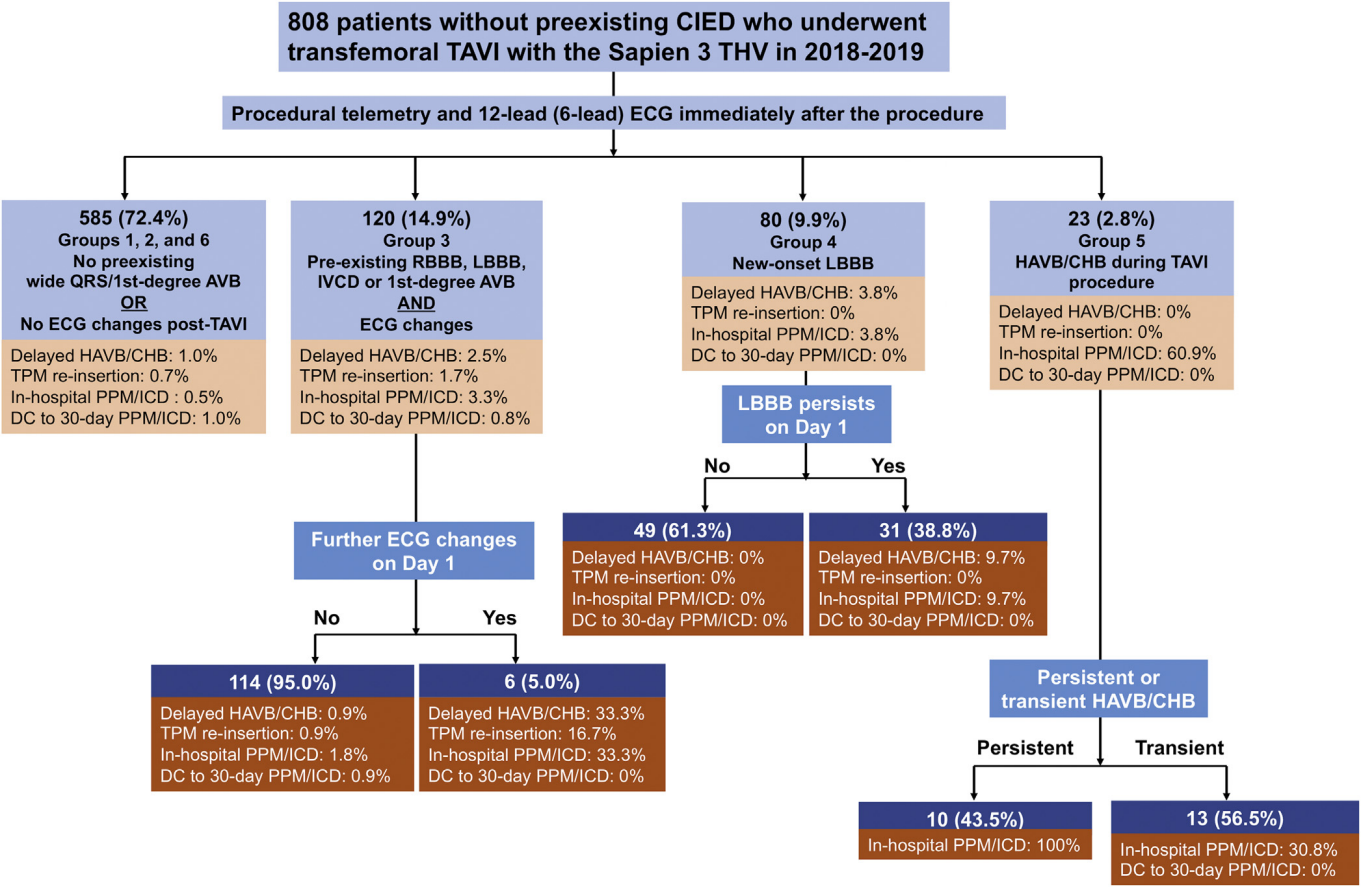
**HAVB/CHB and pacemaker risk in the recategorized groups.** Abbreviations: AVB, atrioventricular block; CHB, complete heart block; CIED, cardiac implantable electronic device; DC, discharge; ECG, electrocardiogram; HAVB, high-degree atrioventricular block; ICD, implantable cardioverter defibrillator; IVCD, interventricular conduction delay; LBBB, left bundle branch block; PPM, permanent pacemaker; RBBB, right bundle branch block; TAVI, transcatheter aortic valve implantation; THV, transcatheter heart valve.

**Figure 4:**
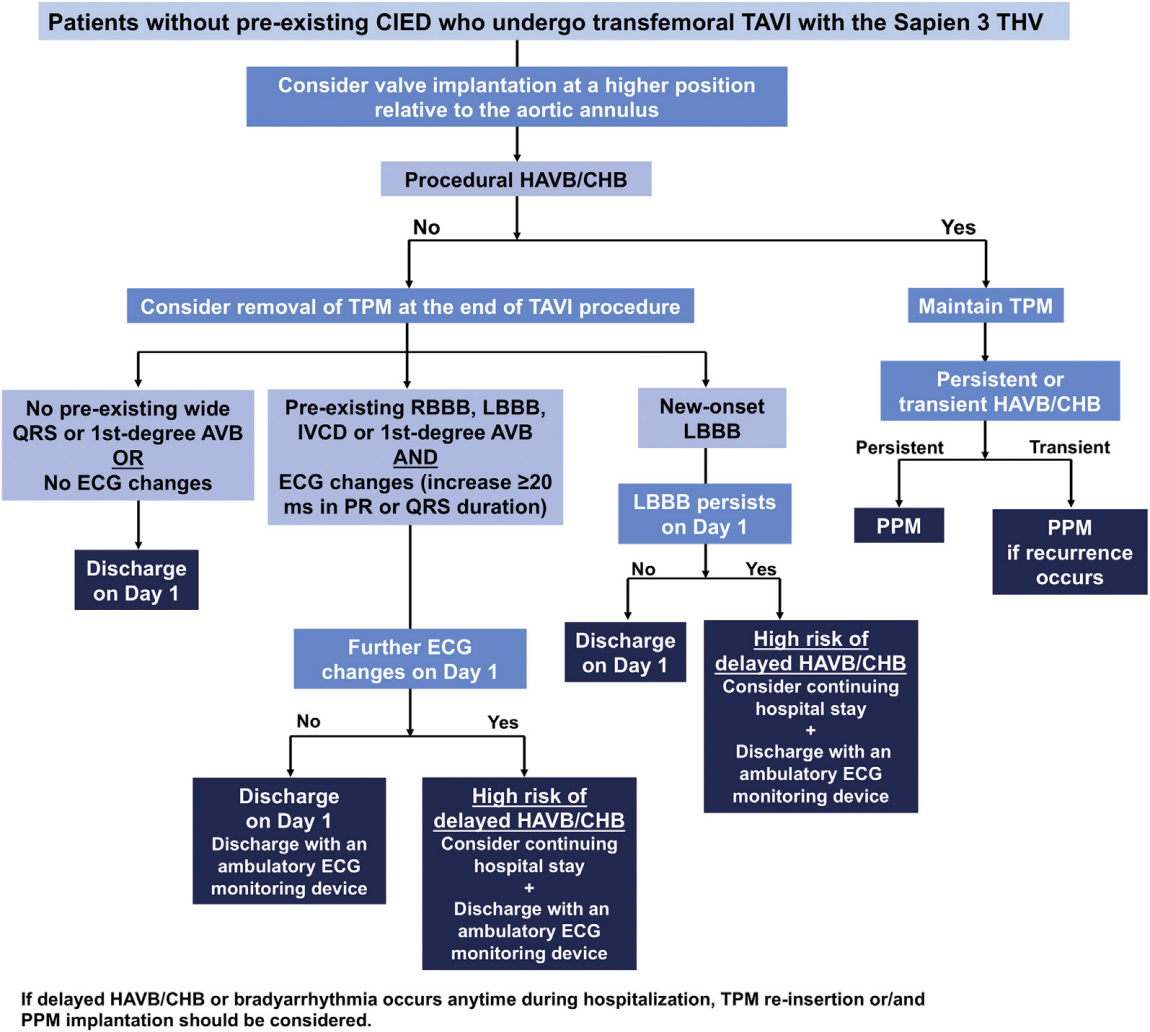
**A proposed modified JACC Scientific Expert Panel algorithm for conduction system disorders in transfemoral TAVI with the Sapien 3 THV.** Abbreviations: AVB, atrioventricular block; CHB, complete heart block; CIED, cardiac implantable electronic device; ECG, electrocardiogram; HAVB, high-degree atrioventricular block; IVCD, interventricular conduction delay; JACC, Journal of the American College of Cardiology; LBBB, left bundle branch block; RBBB, right bundle branch block; TAVI, transcatheter aortic valve implantation; THV, transcatheter heart valve; TPM, temporary pacemaker.

Groups 1, 2, and 6 could be combined into a single group due to a low risk of delayed HAVB/CHB (1.0%: Figure 3). In group 3, patients with further ECG changes (defined as a further increase in PR or QRS duration) on day 1, albeit uncommon (5.0% of patients in group 3), had a much higher delayed HAVB/CHB rate (33.3% vs. 0.9%) than those without further ECG changes. In group 4, the new-onset LBBB resolved on day 1 in 49 of 80 (61.3%) patients, none of whom developed HAVB/CHB; all HAVB/CHB episodes (n = 3) after new-onset LBBB occurred in patients with persistent LBBB on day 1, leading to a relatively high delayed HAVB/CHB rate in this subgroup (9.7% [3/31]). Based on these data, we propose a modification to the 2019 expert panel algorithm for patients who undergo transfemoral TAVI with balloon-expandable valve (Figure 4).

## Discussion

The present analysis has several pertinent findings; (i) the actual timing of TPM removal and hospital discharge were concordant with those of the expert panel recommendations for the majority of patients in group 1, with an infrequent need for TPM reinsertion and PPM implantation; (ii) those recommendations also appeared reasonable in group 5, with high requirement of PPM implantation; (iii) in contrast, in groups 2-4, TPM removal at the end of the procedure (earlier than recommended by the expert panel) was feasible in almost all patients, with a low risk of TPM reinsertion (<2.0%); earlier discharge than recommended was also feasible in the majority (50%-60%) of patients, with a low risk of readmission for PPM implantation (<3.0%); and (iv) further risk stratification of groups 3 and 4 may be possible by using data on ongoing, dynamic ECG changes on post-TAVI day 1.

### Feasibility and Safety of Earlier TPM Removal

In the present study, the TPM reinsertion rate after early TPM removal was low across groups (1.1%). Although a total of 10 patients received PPM/ICD implantation after early TPM removal, 7 out of the 10 PPM/ICD cases did not need TPM reinsertion, suggestive of the fact that the majority of PPM/ICD implantations did not result from the early TPM removal. The expert panel recommends TPM removal immediately after TAVI only in group 1. In contrast, the present study revealed that TPM removal immediately after the procedure was feasible in most TAVI recipients with a low risk of TPM reinsertion (0.0%-1.8%) except for group 5. Delayed HAVB/CHB may manifest emergently with hemodynamic instability in those with early TPM removal after TAVI.^11^, ^12^, ^13^, ^14^, ^15^, ^16^ In the present study, however, the delayed HAVB/CHB rate was much lower (1.5%) than that in other studies (>7%).^11^, ^12^, ^13^ Moreover, delayed HAVB/CHB did not always require TPM reinsertion. A strategy of TPM removal immediately after TAVI, therefore, appears reasonable in all patients except for group 5, the caveat being close, continual monitoring thereafter such that TPM reinsertion is rapidly available upon demand.

### Pre-Existing RBBB

Pre-existing RBBB is the most consistent electrophysiological substrate predisposing to HAVB/CHB following TAVI.^17^, ^18^, ^19^, ^20^, ^21^ The present analysis supports this notion by demonstrating pre-existing RBBB TAVI recipients had a high HAVB/CHB rate (15.7%). However, there is limited evidence supporting the expert panel recommendations of maintaining TPM for 24 ​hours (or overnight) after TAVI in all patients with pre-existing RBBB. While prior studies reported pre-existing RBBB to associate with a higher rate of delayed HAVB/CHB,^11^^,^^12^^,^^14^ no study has yet examined the effectiveness of routinely maintaining a TPM after TAVI in patients with pre-existing RBBB. Notably, the present study revealed that most (88.9%) HAVB/CHB events in patients with pre-existing RBBB occurred during the procedure, which is consistent with a recent Canadian single-center study (86.4%).^15^ Also, the present data are consistent with a recent U.S. single-center study^11^ demonstrating a low sensitivity (17% and 27%, respectively) of pre-existing RBBB in predicting delayed HAVB/CHB. Hence, pre-existing RBBB alone may not be a sufficient reason for “prophylactic” routine maintenance of TPM after TAVI.

### Pre-Existing Conduction Disturbances and Post-TAVI ECG Changes

Owing to scarce data, group 3 represents the most challenging patient cohort regarding TPM and PPM management, as acknowledged by the expert panel,^4^ who cited only 2 studies.^12^^,^^16^ One of them was a Danish single-center study reporting that a PR interval ≥240 ​ms and/or QRS duration ≥150 ​ms immediately after TAVI were associated with a higher risk of delayed HAVB/CHB.^16^ However, the present study demonstrated that a PR interval ≥240 ​ms and/or QRS duration ≥150 ​ms immediately after TAVI had a low sensitivity (42%) and a low positive predictive value (4.7%) in predicting delayed HAVB/CHB (Supplemental Table 7). Given the relatively low delayed HAVB/CHB rate (2.5%) in group 3, the present study suggests that TPM removal immediately after TAVI may be a valid option in selected patients within group 3. Nonetheless, the present study suggests that further ECG changes on day 1 lead to a much higher risk of delayed HAVB/CHB. Thus, we recommend considering a longer hospital stay and discharge with an ambulatory ECG monitoring device in this high-risk subset (Figure 4).

### New-Onset LBBB

Owing to conflicting prior data, the association between new-onset LBBB and delayed HAVB/CHB remains controversial.^11^, ^12^, ^13^, ^14^ Although the expert panel recommends maintaining the TPM in patients with new-onset LBBB (group 4), there currently appears to be a lack of evidence to support this. In the present study, the delayed HAVB/CHB rate in group 4 was 3.8%, which was relatively high compared with that in other groups, but still remained relatively low from an overall clinical perspective, with no case requiring TPM reinsertion. Given the unproven benefit of prolonged TPM insertion in group 4, our data suggest that TPM removal following TAVI in this setting appears safe, however, only in the context of close observation with continuous rhythm monitoring. Importantly, new-onset persistent LBBB on day 1 appears to pose a relatively high risk of delayed HAVB/CHB. A prior ambulatory ECG monitoring study reported that the first bradyarrhythmic episode occurred within 30 days in half of the patients with new-onset persistent LBBB and bradyarrhythmic episodes.^22^ These data highlight the importance of continuous ECG monitoring during the early period after discharge in patients with new-onset persistent LBBB, as the expert panel^4^ and a recent review^23^ recommend.

### Feasibility and Safety of Earlier Discharge After TAVI

Recent studies have demonstrated the feasibility and safety of next-day discharge following transfemoral TAVI.^24^, ^25^, ^26^, ^27^ However, the expert panel does not recommend next-day discharge in groups 2-4. In the present study, next-day discharge was successfully achieved in about 40%-60% of the patients in groups 2-4, with a low 30-day readmission rate for PPM/ICD implantation (<3.0%). Notably, our procedural strategies resulted in a 30-day PPM rate of 3.7% in Sapien 3 recipients, which was lower than that of the recent 3M TAVR study (5.7%) where next-day discharge was applied.^25^ Our finding suggests that safe next-day discharge may be achievable in selected patients of groups 2-4 in a clinical setting with a relatively low PPM risk where our procedural strategies are applicable, as we proposed in our modified algorithm (Figure 4). Further investigations are needed to appropriately select patients suitable for the next-day discharge among patients with pre-existing or new conduction disturbances. The present study also demonstrated that all delayed HAVB/CHB episodes occurred within 8 days after TAVI, which is consistent with prior observations (91%-100%).^11^, ^12^, ^13^ Hence, it is important to provide close follow-up with ambulatory ECG monitoring to selected patients at high risk of delayed HAVB/CHB.

### Study Limitations

Several caveats of the present analysis warrant consideration. First, this study represents the TAVI workflow at a single high-volume center with a low pacemaker rate using a high-implantation technique with the Sapien 3 valve. Hence, the present data may not be generalizable to self-expanding valve recipients or other centers with a higher pacemaker rate or a different implantation technique. The ongoing multicenter, prospective PROMOTE study (NCT04139616) will provide further validations of the expert panel recommendations. Second, although the present study analyzed a large contemporary TAVI cohort, the sample size in each specific group (group 1-6) may be underpowered for subgroup analyses. Third, we were unable to completely capture asymptomatic bradyarrhythmias following hospital discharge due to the absence of routine ambulatory ECG monitoring after discharge in this retrospective study. Lastly, the modified algorithm we propose (Figure 4) was based on our single-center experience and thus requires external validation in a large multicenter cohort.

## Conclusions

The present retrospective study suggests that earlier TPM removal and hospital discharge in a considerably larger number of TAVI recipients than proposed by recent expert panel guidelines are achievable. Next-day discharge after TAVI may safely be adopted in patients with a broader array of pre-existing and new-onset conduction disturbances, wherein we propose a modified conduction system workflow algorithm for transfemoral TAVI recipients with a balloon-expandable valve. While requiring further prospective validation, the present findings provide novel contemporary data to optimize the TAVI workflow in a safe and cost-efficient manner, with potentially significant implications for TAVI practice.

## Ethics Statement

The present research has adhered to the relevant ethical guidelines. The study was approved by the institutional review board of the Cleveland Clinic. Informed consent requirement was waived due to the retrospective nature of the study.

## Funding

This study was made possible by a generous gift from Jennifer and Robert McNeil. The funders had no role in the design and conduct of the study; in the collection, analysis, and interpretation of the data; or in the preparation, review, or approval of the manuscript.

## Disclosure statement

The authors declare no conflict of interest.

## References

[1] Kawsara A., Sulaiman S., Alqahtani F. (2020). Temporal trends in the incidence and outcomes of pacemaker implantation after transcatheter aortic valve replacement in the United States (2012-2017). J Am Heart Assoc.

[2] Lilly S.M., Deshmukh A.J., Epstein A.E. (2020). 2020 ACC expert consensus decision pathway on management of conduction disturbances in patients undergoing transcatheter aortic valve replacement: a report of the American College of Cardiology Solution Set Oversight Committee. J Am Coll Cardiol.

[3] Mazzella A.J., Hendrickson M.J., Arora S. (2021). Shifting trends in timing of pacemaker implantation after transcatheter aortic valve replacement. JACC Cardiovasc Interv.

[4] Rodes-Cabau J., Ellenbogen K.A., Krahn A.D. (2019). Management of conduction disturbances associated with transcatheter aortic valve replacement: JACC Scientific Expert Panel. J Am Coll Cardiol.

[5] Malebranche D., Bartkowiak J., Ryffel C. (2021). Validation of the 2019 expert consensus algorithm for the management of conduction disturbances after TAVR. JACC Cardiovasc Interv.

[6] Carroll J.D., Mack M.J., Vemulapalli S. (2020). STS-ACC TVT registry of transcatheter aortic valve replacement. J Am Coll Cardiol.

[7] Kappetein A.P., Head S.J., Genereux P. (2012). Updated standardized endpoint definitions for transcatheter aortic valve implantation: the Valve Academic Research Consortium-2 consensus document. J Am Coll Cardiol.

[8] Sammour Y., Banerjee K., Kumar A. (2021). Systematic approach to high implantation of SAPIEN-3 valve achieves a lower rate of conduction abnormalities including pacemaker implantation. Circ Cardiovasc Interv.

[9] Surawicz B., Childers R., Deal B.J. (2009). AHA/ACCF/HRS recommendations for the standardization and interpretation of the electrocardiogram: part III: intraventricular conduction disturbances: a scientific statement from the American Heart Association Electrocardiography and Arrhythmias Committee, Council on Clinical Cardiology; the American College of Cardiology Foundation; and the Heart Rhythm Society. Endorsed by the International Society for Computerized Electrocardiology. J Am Coll Cardiol.

[10] Kusumoto F.M., Schoenfeld M.H., Barrett C. (2019). 2018 ACC/AHA/HRS guideline on the evaluation and management of patients with bradycardia and cardiac conduction delay: a report of the American College of Cardiology/American Heart Association Task Force on clinical practice guidelines and the Heart Rhythm Society. J Am Coll Cardiol.

[11] Ream K., Sandhu A., Valle J. (2019). Ambulatory rhythm monitoring to detect late high-grade atrioventricular block following transcatheter aortic valve replacement. J Am Coll Cardiol.

[12] Mangieri A., Lanzillo G., Bertoldi L. (2018). Predictors of advanced conduction disturbances requiring a late (>/=48 H) permanent pacemaker following transcatheter aortic valve replacement. JACC Cardiovasc Interv.

[13] Toggweiler S., Stortecky S., Holy E. (2016). The electrocardiogram after transcatheter aortic valve replacement determines the risk for post-procedural high-degree AV block and the need for telemetry monitoring. JACC Cardiovasc Interv.

[14] Tian Y., Padmanabhan D., McLeod C.J. (2019). Utility of 30-day continuous ambulatory monitoring to identify patients with delayed occurrence of atrioventricular block after transcatheter aortic valve replacement. Circ Cardiovasc Interv.

[15] Muntane-Carol G., Del Val D., Junquera L. (2020). Timing and evolution of advanced conduction disturbances in patients with right bundle branch block undergoing transcatheter aortic valve replacement. Europace.

[16] Jorgensen T.H., De Backer O., Gerds T.A., Bieliauskas G., Svendsen J.H., Sondergaard L. (2018). Immediate post-procedural 12-lead electrocardiography as predictor of late conduction defects after transcatheter aortic valve replacement. JACC Cardiovasc Interv.

[17] Jilaihawi H., Zhao Z., Du R. (2019). Minimizing permanent pacemaker following repositionable self-expanding transcatheter aortic valve replacement. JACC Cardiovasc Interv.

[18] Maeno Y., Abramowitz Y., Kawamori H. (2017). A highly predictive risk model for pacemaker implantation after TAVR. JACC Cardiovasc Imaging.

[19] Mauri V., Reimann A., Stern D. (2016). Predictors of permanent pacemaker implantation after transcatheter aortic valve replacement with the SAPIEN 3. JACC Cardiovasc Interv.

[20] Kiani S., Kamioka N., Black G.B. (2019). Development of a risk score to predict new pacemaker implantation after transcatheter aortic valve replacement. JACC Cardiovasc Interv.

[21] Nazif T.M., Dizon J.M., Hahn R.T. (2015). Predictors and clinical outcomes of permanent pacemaker implantation after transcatheter aortic valve replacement: the PARTNER (Placement of AoRtic TraNscathetER Valves) trial and registry. JACC Cardiovasc Interv.

[22] Rodes-Cabau J., Urena M., Nombela-Franco L. (2018). Arrhythmic burden as determined by ambulatory continuous cardiac monitoring in patients with new-onset persistent left bundle branch block following transcatheter aortic valve replacement: the MARE study. JACC Cardiovasc Interv.

[23] Muntane-Carol G., Philippon F., Nault I. (2021). Ambulatory electrocardiogram monitoring in patients undergoing transcatheter aortic valve replacement: JACC state-of-the-art review. J Am Coll Cardiol.

[24] Yerasi C., Tripathi B., Wang Y. (2021). National trends and 30-day readmission rates for next-day-discharge transcatheter aortic valve replacement: an analysis from the Nationwide Readmissions Database, 2012-2016. Am Heart J.

[25] Wood D.A., Lauck S.B., Cairns J.A. (2019). The Vancouver 3M (multidisciplinary, multimodality, but minimalist) clinical pathway facilitates safe next-day discharge home at low-, medium-, and high-volume transfemoral transcatheter aortic valve replacement centers: the 3M TAVR study. JACC Cardiovasc Interv.

[26] Moriyama N., Vento A., Laine M. (2019). Safety of next-day discharge after transfemoral transcatheter aortic valve replacement with a self-expandable versus balloon-expandable valve prosthesis. Circ Cardiovasc Interv.

[27] Kamioka N., Wells J., Keegan P. (2018). Predictors and clinical outcomes of next-day discharge after minimalist transfemoral transcatheter aortic valve replacement. JACC Cardiovasc Interv.

