# Supplementary material for: Conduction Disturbance, Pacemaker Rates, and Hospital Length of Stay Following Transcatheter Aortic Valve Implantation with the Sapien 3 Valve
Source: Struct Heart. 2022 Mar 30;6(3):100019. doi: 10.1016/j.shj.2022.100019 (PMC10236805; doi:10.1016/j.shj.2022.100019)
Supplement: Supplemental Figures 1–4 and Tables 1–7 [file mmc1.docx]

**eFigure 1. High deployment techniques with the Sapien 3 valve**

Our high deployment technique is based upon the notion of simply isolating the non-coronary cusp in the right anterior oblique (RAO) caudal imaging plane (similar to the right-left cusp overlap technique) to identify the annular plane. In the context of deploying the Sapien 3 balloon-expandable transcatheter heart valve (THV) system, upon annular engagement/valve crossing, the C-arm/image intensifier is rotated to remove the parallax from the inflow of the valve stent frame (Figures A-D). This elucidates a line of radiographic lucency just above the stent frame inflow, corresponding to the vertical depth and position of where the valve inflow will ultimately foreshorten upon full expansion (6). This technique allows one to accurately predict as well as actively modify the valve depth positioning during deployment.

**Sapien 3**

**
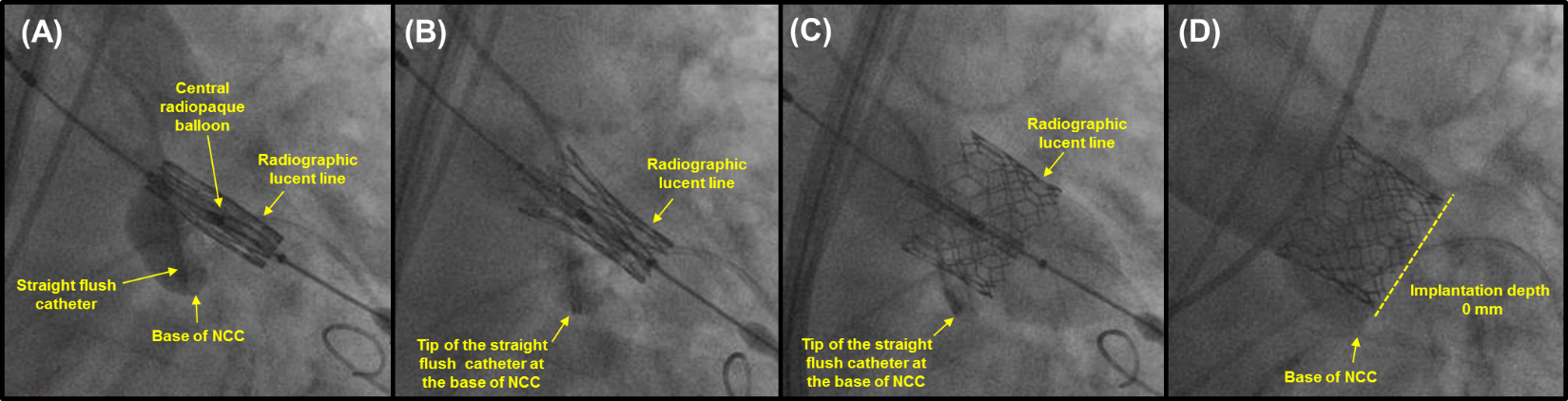
**

(A) A straight flush catheter is placed in the non-coronary cusp (NCC) to mark the base of NCC and obtain an aortogram. Sapien 3 THV is positioned by aligning the radiographic lucent line at the base of NCC in the RAO caudal view that remove the parallax from the inflow of the valve stent frame; (B) and (C) The valve is deployed while maintaining the radiographic lucent line at the base of the NCC during the valve expansion; (D) Final aortography allows measurement of the valve implantation depth, which shows 0 mm relative to the base of the NCC in this case.

**eFigure 2. Selection of study patients**

**
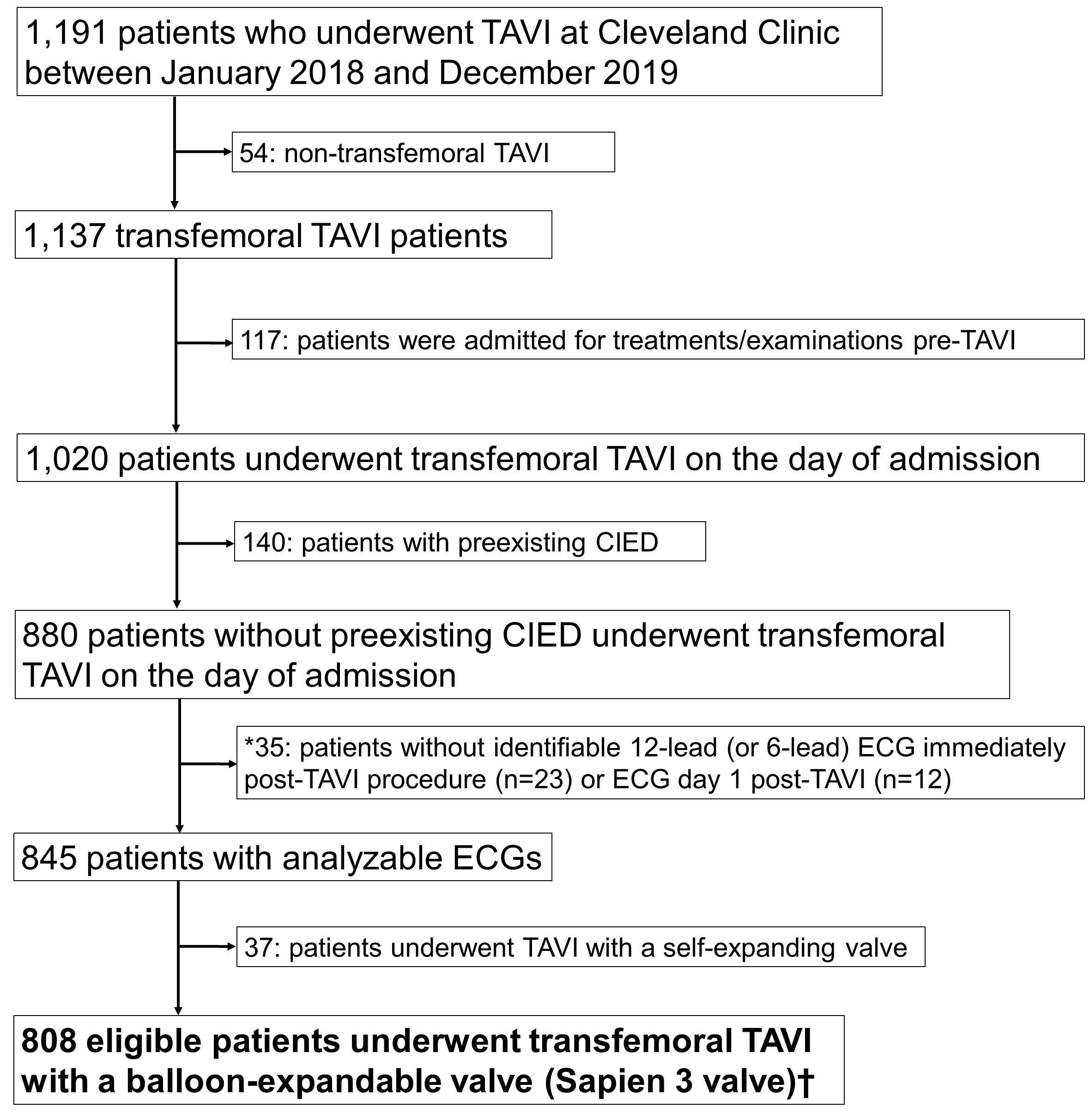
**

*None of the 35 patients developed procedural or delayed HAVB/CHB, underwent any CIED implantation, or died within 180 days post-TAVI. †All balloon-expandable valves used at our institution in the study period were the Sapien 3 valve.

CHB = complete heart block; CIED = cardiac implantable electronic device; ECG = electrocardiogram; HAVB = high-degree atrioventricular block; TAVI = transcatheter aortic valve implantation.

**eFigure 3. Distributions of actual and proposed timing of TPM removal after TAVI**


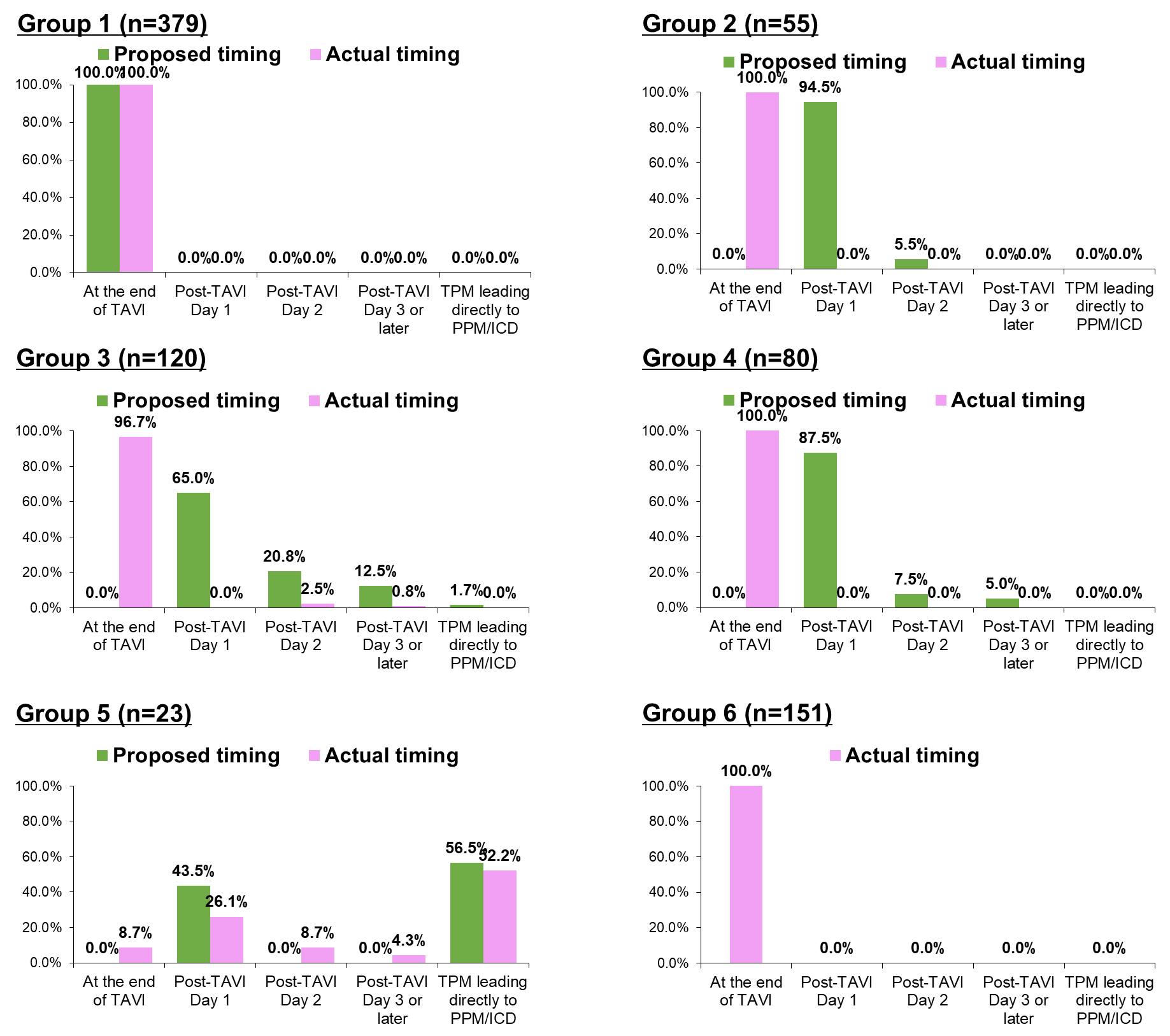


ICD = implantable cardioverter defibrillator; PPM = permanent pacemaker; TAVI = transcatheter aortic valve implantation; TPM = temporary pacemaker.

**eFigure 4. Distributions of actual and proposed timing of hospital discharge after TAVI with the Sapien 3 valve**


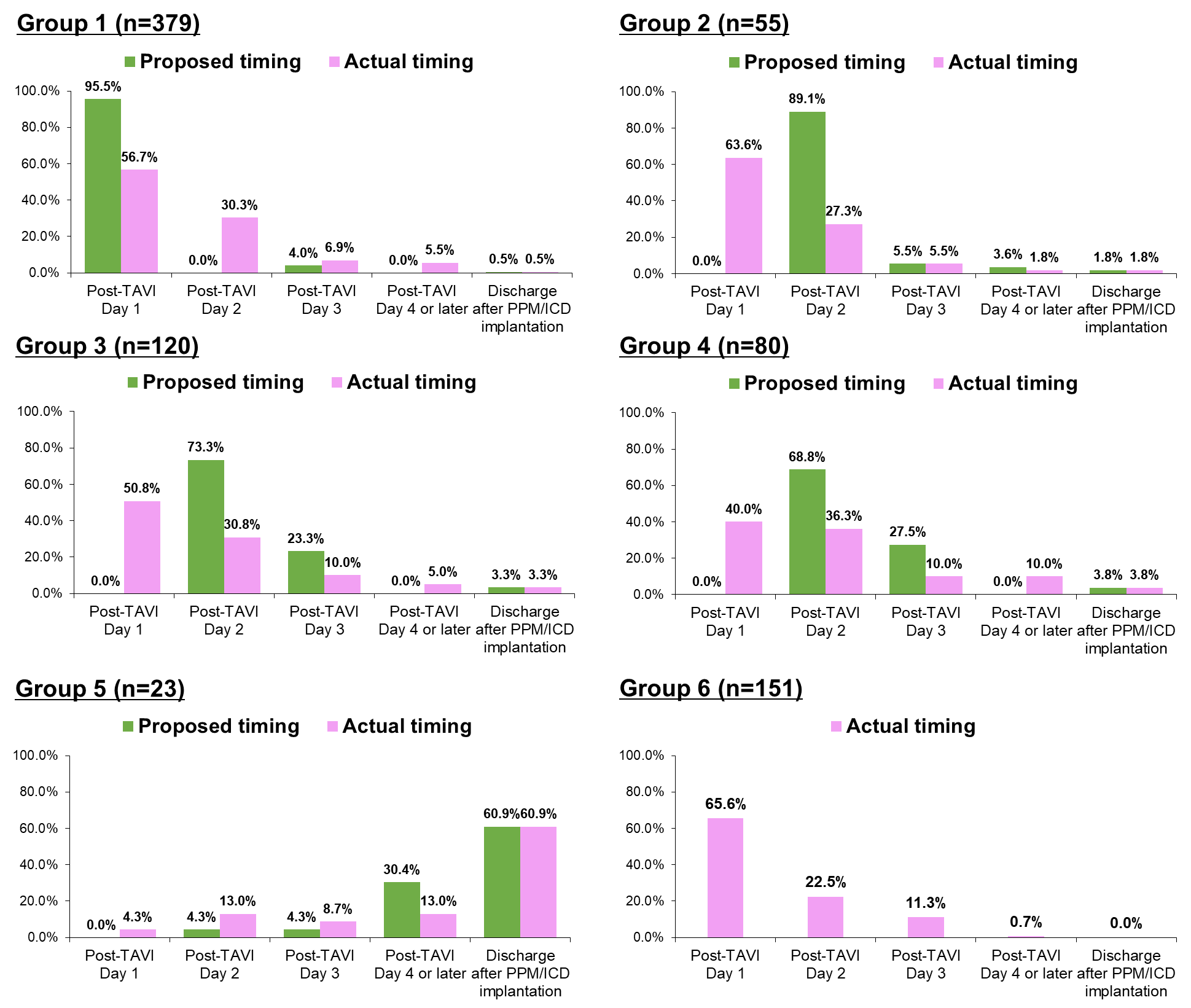


ICD = implantable cardioverter defibrillator; PPM = permanent pacemaker; TAVI = transcatheter aortic valve implantation.

**eTable 1. In-hospital adverse events and discharge status**

|  | **Groups 1-6** | **Group 1 (No ECG changes without pre-existing RBBB)** | **Group 2 (No ECG changes with pre-existing RBBB)** | **Group 3 (ECG changes with pre-existing conduction disturbance*)** | **Group 4 (New-onset LBBB)** | **Group 5 (HAVB/CHB during the procedure)** | **Group 6 (ECG changes without pre-existing conduction disturbance* and without new-onset LBBB or HVB/CHB)** |  |
| --- | --- | --- | --- | --- | --- | --- | --- | --- |
|  | **(N=808)** | **(n=379)** | **(n=55)** | **(n=120)** | **(n=80)** | **(n=23)** | **(n=151)** | **P value** |
| **In-hospital adverse events and length of stay** |  |  |  |  |  |  |  |  |
| Stroke/TIA | 6 (0.7) | 4 (1.1) | 1 (1.8) | 1 (0.8) | 0 (0.0) | 0 (0.0) | 0 (0.0) | 0.57 |
| Acute kidney injury | 27 (3.3) | 14 (3.7) | 0 (0.0) | 2 (1.7) | 1 (1.3) | 2 (8.7) | 8 (5.3) | 0.15 |
| Major vascular complication | 4 (0.5) | 3 (0.8) | 0 (0.0) | 0 (0.0) | 0 (0.0) | 0 (0.0) | 1 (0.7) | 1.00 |
| Minor vascular complication | 89 (11.0) | 47 (12.4) | 3 (5.5) | 9 (7.5) | 7 (8.8) | 3 (13) | 20 (13.2) | 0.39 |
| New-onset atrial fibrillation | 15 (1.9) | 7 (1.8) | 3 (5.5) | 2 (1.7) | 2 (2.5) | 0 (0.0) | 1 (0.7) | 0.34 |
| Myocardial infarction | 0 (0.0) | 0 (0.0) | 0 (0.0) | 0 (0.0) | 0 (0.0) | 0 (0.0) | 0 (0.0) | – |
| Coronary obstruction | 3 (0.4) | 2 (0.5) | 0 (0.0) | 1 (0.8) | 0 (0.0) | 0 (0.0) | 0 (0.0) | 0.77 |
| Cardiac tamponade | 3 (0.4) | 1 (0.3) | 0 (0.0) | 1 (0.8) | 0 (0.0) | 0 (0.0) | 1 (0.7) | 0.68 |
| SDU or ICU stay, hours, median (IQR) | 24.0  (23.1, 24.7) | 24.0  (23.0, 24.6) | 24.0  (23.0, 24.4) | 24.1  (23.2, 25.0) | 23.9  (23.0, 24.7) | 24.5  (23.6, 25.2) | 24.1  (23.2, 24.7) | 0.36 |
| Length of stay, days, median (IQR) | 1 (1, 2) | 1 (1, 2) | 1 (1, 2) | 1 (1, 2) | 2 (1, 2) | 4 (2.5, 4.5) | 1 (1, 2) | <0.001 |
| **Discharge status** |  |  |  |  |  |  |  |  |
| Death | 0 (0.0) | 0 (0.0) | 0 (0.0) | 0 (0.0) | 0 (0.0) | 0 (0.0) | 0 (0.0) | – |
| Discharge with Zio Patch monitoring (N=784)† | 86 (11.0) | 42 (11.1) | 4 (7.4) | 12 (10.3) | 14 (18.2) | 4 (44.4) | 10 (6.6) | 0.009 |
| Discharge home | 768 (95.0) | 360 (95.0) | 54 (98.2) | 115 (95.8) | 76 (95.0) | 21 (91.3) | 142 (94.0) | 0.76 |

Values are n (%) unless otherwise indicated. *Conduction disturbance includes RBBB, LBBB, interventricular conduction delay or/and 1st degree atrioventricular block. †Includes patients discharged without in-hospital PPM/ICD implantation. CHB = complete heart block; ECG = electrocardiogram; HAVB = high-degree atrioventricular block; ICD = implantable cardioverter defibrillator; ICU = intensive care unit; IQR = interquartile range; LBBB = left bundle branch block; PPM = permanent pacemaker; RBBB = right bundle branch block; SDU = step-down unit; TIA = transient ischemic attack.

**eTable 2. Detailed ECG findings and post-TAVI events**

|  | **Groups 1-6** | **Group 1 (No ECG changes without pre-existing RBBB)** | **Group 2 (No ECG changes with pre-existing RBBB)** | **Group 3 (ECG changes with pre-existing conduction disturbance*****)** | **Group 4 (New-onset LBBB)** | **Group 5 (HAVB/CHB during the procedure)** | **Group 6 (ECG changes without pre-existing conduction disturbance*** **and without new-onset LBBB or HVB/CHB)** |  | |
| --- | --- | --- | --- | --- | --- | --- | --- | --- | --- |
|  | **(N=808)** | **(n=379)** | **(n=55)** | **(n=120)** | **(n=80)** | **(n=23)** | **(n=151)** | **P value** | |
| **Pre-TAVI baseline ECG findings** | | | | | | | | | |
| Rhythm |  |  |  |  |  |  |  | <0.001 | |
| Sinus rhythm | 681 (84.3) | 283 (74.7) | 48 (87.3) | 117 (97.5) | 72 (90.0) | 18 (78.3) | 143 (94.7) |  | |
| Atrial fibrillation | 104 (12.9) | 82 (21.6) | 6 (10.9) | 2 (1.7) | 5 (6.3) | 3 (13.0) | 6 (4.0) |  | |
| Atrial flutter | 16 (2.0) | 9 (2.4) | 1 (1.8) | 0 (0.0) | 3 (3.8) | 1 (4.3) | 2 (1.3) |  | |
| Junctional rhythm | 7 (0.9) | 5 (1.3) | 0 (0.0) | 1 (0.8) | 0 (0.0) | 1 (4.3) | 0 (0.0) |  | |
| Heart rate, beat/m | 68 (60, 78) | 68 (60, 78) | 64 (59, 79) | 67 (60.5, 77.5) | 68 (59.5, 76) | 77 (62.5, 86) | 68 (61, 78) | 0.26 | |
| LBBB | 45 (5.6) | 29 (7.7) | 0 (0.0) | 15 (12.5) | 0 (0.0) | 1 (4.3) | 0 (0.0) | <0.001 | |
| Non-specific IVCD | 38 (4.7) | 23 (6.1) | 0 (0.0) | 8 (6.7) | 7 (8.8) | 0 (0.0) | 0 (0.0) | 0.001 | |
| **Post-TAVI** |  |  |  |  |  |  |  |  | |
| TPM removal at the end of TAVI | 783 (96.9) | 379 (100.0) | 55 (100.0) | 116 (96.7) | 80 (100.0) | 2 (8.7) | 151 (100.0) | <0.001 | |
| ECG change**:** end of TAVI - Pre-TAVI | | | | | | | | | |
| ΔPR interval, ms (N=643)† | +16 (+4, +28) | +8 (+0, +14) | +6 (-8, +14) | +32 (+25, +47) | +16 (+2, +30) | +74 (+45, +98) | +28 (+22, +38) | <0.001 | |
| ΔQRS duration, ms (N=792)‡ | +4 (-2, +12) | +2 (-2, +6) | +0 (-7, +6) | +2 (-2, +10) | +51 (+40, +60) | +4 (+2, +10) | +6 (+0, +18) | <0.001 | |
| ECG change: Day 1 - end of TAVI | | | | | | | | | |
| ΔPR interval, ms (N=644)§ | -16 (-28, -8) | -10 (-18, -4) | -10 (-18, -4) | -30 (-54, -18) | -14 (-28, -6) | -56 (-90, -37) | -22 (-32, -12) | <0.001 | |
| ΔQRS duration, ms (N=791)\|\| | -2 (-8, +2) | -2 (-6, +2) | 0 (-6, +4) | -2 (-8, +2) | -32 (-46, -6) | 0 (-2, +1) | -4 (-14, 0) | <0.001 | |
| **Events during hospitalization** | | | | | | | | |  |
| HAVB/CHB | 34 (4.2) | 4 (1.1) | 1 (1.8) | 3 (2.5) | 3 (3.8) | 23 (100.0) | 0 (0.0) | <0.001 | |
| Procedural HAVB/CHB | 23 (2.8) | 0 (0.0) | 0 (0.0) | 0 (0.0) | 0 (0.0) | 23 (100.0) | 0 (0.0) | <0.001 | |
| Delayed HAVB/CHB | 11 (1.4) | 4 (1.1) | 1 (1.8) | 3 (2.5) | 3 (3.8) | 0 (0.0) | 0 (0.0) | 0.13 | |
| TPM re-insertion | 6 (0.7) | 3 (0.8) | 1 (1.8) | 2 (1.7) | 0 (0.0) | 0 (0.0) | 0 (0.0) | 0.40 | |
| PPM/ICD implantation | 24 (3.0) | 2 (0.5) | 1 (1.8) | 4 (3.3) | 3 (3.8) | 14 (60.9) | 0 (0.0) | <0.001 | |
| Death | 0 (0.0) | 0 (0.0) | 0 (0.0) | 0 (0.0) | 0 (0.0) | 0 (0.0) | 0 (0.0) | <0.001 | |
| **Events during the period between discharge and 30 days after TAVI** | | | | | | | | | |
| HAVB/CHB | 2 (0.2) | 1 (0.3) # | 1 (1.8) | 0 (0.0) | 0 (0.0) | 0 (0.0) | 0 (0.0) | 0.37 | |
| PPM/ICD implantation | 7 (0.9) | 3 (0.8) | 2 (3.6) | 1 (0.8) | 0 (0.0) | 0 (0.0) | 1 (0.7) | 0.39 | |
| Death | 0 (0.0) | 0 (0.0) | 0 (0.0) | 0 (0.0) | 0 (0.0) | 0 (0.0) | 0 (0.0) | – | |
| **Events during the period between 31 days and 180 days after TAVI** | | | | | | | | | |
| PPM/ICD implantation | 8 (1.0) | 2 (0.5) | 0 (0.0) | 4 (3.3) | 1 (1.3) | 0 (0.0) | 1 (0.7) | 0.17 | |
| Death | 15 (1.9) | 7 (1.8) | 0 (0.0) | 3 (2.5) | 2 (2.5) | 1 (4.3) | 2 (1.3) | 0.65 | |

Values are n (%), n/total n (%), or median (interquartile range). No patient died during index hospitalization or within 30 days after TAVI. *Conduction disturbance includes RBBB, LBBB, interventricular conduction delay or/and 1st degree atrioventricular block. †Includes patients with sinus rhythm in both ECGs pre-TAVI and at the end of TAVI. ‡Includes patients without ventricular-paced rhythm at the end of TAVI. §Includes patients with sinus rhythm on both ECGs at the end of TAVI and Day 1. ||Includes patients without ventricular-paced rhythm on either ECGs at the end of TAVI or Day 1. #This patient developed post-discharge recurrent HAVB/CHB after experiencing transient CHB on the same day of TAVI. AVB = atrioventricular block; CHB = complete heart block; ECG = electrocardiogram; HAVB = high-degree atrioventricular block; ICD = implantable cardioverter defibrillator; IVCD = interventricular conduction delay; LBBB = left bundle branch block; PPM = permanent pacemaker; RBBB = right bundle branch block; TAVI = transcatheter aortic valve implantation; TPM = temporary pacemaker.

**eTable 3. Details on post-TAVI conduction disturbances and device implantation**

|  | **Groups 1-6** | **Group 1 (No ECG changes without pre-existing RBBB)** | **Group 2 (No ECG changes with pre-existing RBBB)** | **Group 3 (ECG changes with pre-existing conduction disturbance)** | **Group 4 (New-onset LBBB)** | **Group 5 (HAVB/CHB during the procedure)** | **Group 6 (ECG changes without pre-existing conduction disturbance and without new-onset LBBB or HVB/CHB)** |  |
| --- | --- | --- | --- | --- | --- | --- | --- | --- |
|  | **(N=808)** | **(n=379)** | **(n=55)** | **(n=120)** | **(n=80)** | **(n=23)** | **(n=151)** | **P value** |
| **Events during hospitalization** | | | | | | | | |
| HAVB/CHB | 34 (4.2) | 4 (1.1) | 1 (1.8) | 3 (2.5) | 3 (3.8) | 23 (100.0) | 0 (0.0) | <0.001 |
| HAVB | 5 (0.6) | 2 (0.5) | 0 (0.0) | 2 (1.7) | 1 (1.3) | 0 (0.0) | 0 (0.0) | 0.43 |
| CHB | 29 (3.6) | 2 (0.5) | 1 (1.8) | 1 (0.8) | 2 (2.5) | 23 (100.0) | 0 (0.0) | <0.001 |
| Transient | 18 (2.2) | 2 (0.5) | 1 (1.8) | 0 (0.0) | 2 (2.5) | 13 (56.5) | 0 (0.0) | <0.001 |
| Persistent | 11 (1.4) | 0 (0.0) | 0 (0.0) | 1 (0.8) | 0 (0.0) | 10 (43.5) | 0 (0.0) | <0.001 |
| TPM re-insertion | 6 (0.7) | 3 (0.8) | 1 (1.8) | 2 (1.7) | 0 (0.0) | 0 (0.0) | 0 (0.0) | 0.40 |
| Reason for TPM re-insertion | Transient CHB (n=3), Persistent CHB (n=1), HAVB (n=2) | Transient CHB (n=2), HAVB (n=1) | Transient CHB (n=1) | Persistent CHB (n=1), HAVB (n=1) | – | – | – | – |
| PPM/ICD implantation | 24 (3.0) | 2 (0.5) | 1 (1.8) | 4 (3.3) | 3 (3.8) | 14 (60.9) | 0 (0.0) | <0.001 |
| Indication for PPM/ICD | HAVB/CHB (n=21), SSS (n=3) | SSS (n=2) | HAVB/CHB (n=1) | HAVB/CHB (n=3), SSS (n=1) | HAVB/CHB (n=3) | HAVB/CHB (n=14) | – | – |
| Type of device | Dual-chamber PPM (n=17), CRT-P (n=2), CRT-D (n=3), Single-chamber PPM (n=2) | Dual-chamber PPM (n=2) | Dual-chamber PPM (n=1) | Dual-chamber PPM (n=4) | Dual-chamber PPM (n=3) | Dual-chamber PPM (n=7), CRT-P (n=2), CRT-D (n=3), Single-chamber PPM (n=2) | – | – |
| **Events during the period between discharge and 30 days after TAVI** | | | | | | | | |
| HAVB/CHB | 2 (0.2)  (1 new, 1 recurrent) | 1 (0.3)* (recurrent) | 1 (1.8) | 1 (1.7) (new) | 0 (0.0) | 0 (0.0) | 0 (0.0) | 0.37 |
| PPM/ICD implantation | 7 (0.9) | 3 (0.8) | 2 (3.6) | 1 (0.8) | 0 (0.0) | 0 (0.0) | 1 (0.7) | 0.39 |
| Indication for PPM/ICD | HAVB/CHB (n=2), SSS (n=2), LBBB + low LVEF (n=2), AF with slow rate response and bifascicular block† (n=1) | HAVB/CHB (n=1), SSS (n=1), LBBB + low LVEF (n=1), | HAVB/CHB (n=1), AF with slow rate response and fascicular block† (n=1) | LBBB + low LVEF (n=1) | – | – | SSS (n=1) | – |
| Type of device | Dual-chamber PPM (n=4), CRT-D (n=2), Single-chamber PPM (n=1) | Dual-chamber PPM (n=2), CRT-D (n=1) | Dual-chamber PPM (n=1), Single-chamber PPM (n=1) | CRT-D (n=1) | – | – | Dual-chamber PPM (n=1) | – |
| **Events during the period between 31 days and 180 days after TAVI§** | | | | | | | | |
| PPM/ICD implantation | 8 (1.0) | 2 (0.5) | 0 (0.0) | 4 (3.3) | 1 (1.3) | 0 (0.0) | 1 (0.7) | 0.17 |
| Indication for PPM/ICD | HAVB/CHB (n=4), SSS (n=1), LBBB + low LVEF (n=1), SSS and LBBB + low LVEF (n=1), VF (n=1) | LBBB + low LVEF (n=1), SSS and LBBB + low LVEF (n=1) | – | HAVB/CHB (n=2), SSS (n=1), VF (n=1) | HAVB/CHB (n=1) | – | HAVB/CHB (n=1) | – |
| Type of device | Dual-chamber PPM (n=5), CRT-D (n=3) | CRT-D (n=2) | – | Dual-chamber PPM (n=3), CRT-D (n=1) | Dual-chamber PPM (n=1) | – | Dual-chamber PPM (n=1) | – |

Values are n (%) unless otherwise indicated. *This patient developed post-discharge recurrent HAVB/CHB after experiencing transient CHB on the same day of TAVI. †The patient developed syncope without other identifiable cause. AF = atrial fibrillation; CHB = complete heart block; CRT-D = cardiac resynchronization therapy defibrillator; CRT-P = cardiac resynchronization therapy pacemaker; ICD = implantable cardioverter defibrillator; IQR = interquartile range; HAVB = high-degree atrioventricular block; LBBB = left bundle branch block; LVEF = left ventricular ejection fraction; PPM = permanent pacemaker; SSS = sick sinus syndrome; TAVI = transcatheter aortic valve implantation; TPM = temporary pacemaker; VF = ventricular fibrillation.

**eTable 4. Reasons for delayed discharge relative to Expert Panel recommendations**

|  | **Groups 1-5** | **Group 1 (No ECG changes without pre-existing RBBB)** | **Group 2 (No ECG changes with pre-existing RBBB)** | **Group 3 (ECG changes with pre-existing conduction disturbance)** | **Group 4 (New-onset LBBB)** | **Group 5 (HAVB/CHB during the procedure)** |
| --- | --- | --- | --- | --- | --- | --- |
| **No. of patients with delayed discharge compared with proposed timing of discharge** | 183 | 153 | 3 | 13 | 14 | **0** |
| **Main reason for delayed discharge** |  |  |  |  |  |  |
| Routine observation* | 50 (27.3) | 50 (32.7) | 0 (0.0) | 0 (0.0) | 0 (0.0) | – |
| Conduction disturbance or bradycardia | 22 (12.0) | 12 (7.8) | 0 (0.0) | 3 (23.1) | 7 (50) | – |
| Access site issue | 15 (8.2) | 9 (5.9) | 1 (33.3) | 1 (7.7) | 4 (28.6) | – |
| Hemodynamic change, heart failure, or fluid overload | 16 (8.7) | 12 (7.8) | 0 (0.0) | 3 (23.1) | 1 (7.1) | – |
| Medication adjustment | 16 (8.7) | 15 (9.8) | 1 (33.3) | 0 (0.0) | 0 (0.0) | – |
| Other non-cardiac symptoms or complications | 15 (8.2) | 10 (6.5) | 0 (0.0) | 5 (38.5) | 0 (0.0) | – |
| Tachyarrhythmia | 13 (7.1) | 12 (7.8) | 0 (0.0) | 1 (7.7) | 0 (0.0) | – |
| Thrombocytopenia or anemia | 9 (4.9) | 8 (5.2) | 0 (0.0) | 0 (0.0) | 1 (7.1) | – |
| Fever or infection | 7 (3.8) | 7 (4.6) | 0 (0.0) | 0 (0.0) | 0 (0.0) | – |
| Observation due to very old age or multiple comorbidities | 5 (2.7) | 5 (3.3) | 0 (0.0) | 0 (0.0) | 0 (0.0) |  |
| Logistic reason | 5 (2.7) | 4 (2.6) | 0 (0.0) | 0 (0.0) | 1 (7.1) | – |
| Renal function follow-up or dialysis | 4 (2.2) | 3 (2) | 1 (33.3) | 0 (0.0) | 0 (0.0) | – |
| Stroke or TIA | 3 (1.6) | 3 (2) | 0 (0.0) | 0 (0.0) | 0 (0.0) | – |
| Other valve-related complication† | 3 (1.6) | 3 (2) | 0 (0.0) | 0 (0.0) | 0 (0.0) | – |

Values are n (%). *In our institution, all patients who underwent TAVI before August 2018 remained in hospital at least until post-TAVI Day 2 as a part of routine practice. †Follow-up after percutaneous coronary intervention for coronary obstruction (n=2) and computed tomographic evaluation of elevated aortic valve gradients detected on post-TAVI Day 1 echocardiography (n=1). TAVI = transcatheter aortic valve implantation; TIA = transient ischemic attack.

**eTable 5. PPM/ICD risk after TPM removal at the end of TAVI procedure and after hospital discharge on post-TAVI Day 1**

|  | **Groups 1-6** | **Group 1 (No ECG changes without pre-existing RBBB)** | **Group 2 (No ECG changes with pre-existing RBBB)** | **Group 3 (ECG changes with pre-existing conduction disturbance)** | **Group 4 (New-onset LBBB)** | **Group 5 (HAVB/CHB during the procedure)** | **Group 6 (ECG changes without pre-existing conduction disturbance and without new-onset LBBB or HVB/CHB)** | **P value** |
| --- | --- | --- | --- | --- | --- | --- | --- | --- |
| **No. of patients whose TPM was removed at the end of TAVI procedure** | **783** | **379** | **55** | **116** | **80** | **2** | **151** |  |
| Delayed HAVB/CHB during hospitalization | 10 (1.3) | 4 (1.1) | 1 (1.8) | 2 (1.7) | 3 (3.8) | 0 (0.0) | 0 (0.0) | 0.13 |
| TPM re-insertion | 6 (0.8) | 3 (0.8) | 1 (1.8) | 2 (1.7) | 0 (0.0) | 0 (0.0) | 0 (0.0) | 0.30 |
| Resulting in PPM/ICD implantation | 3 (0.4) | 0 (0.0) | 1 (1.8) | 2 (1.7) | – | – | – | – |
| No TPM re-insertion, but requiring PPM/ICD implantation | 8 (1.0) | 2 (0.5) | 0 (0.0) | 1 (0.9) | 3 (3.8) | 2 (100.0) | 0 (0.0) | <0.001 |
| In-hospital PPM/ICD implantation, total | 11 (1.4) | 2 (0.5) | 1 (1.8) | 3 (2.6) | 3 (3.8) | 2 (100.0) | 0 (0.0) | <0.001 |
| In-hospital death | 0 (0.0) | 0 (0.0) | 0 (0.0) | 0 (0.0) | 0 (0.0) | 0 (0.0) | 0 (0.0) | – |
| **No. of patients discharged on post-TAVI Day 1 without PPM/ICD** | **443** | **215** | **35** | **61** | **32** | **1** | **99** |  |
| Discharge to 30-day PPM/ICD implantation | 3 (0.7) | 0 (0.0) | 1 (2.9) | 1 (1.6) | 0 (0.0) | 0 (0.0) | 1 (1.0) | 0.14 |
| Discharge to 30-day death | 0 (0.0) | 0 (0.0) | 0 (0.0) | 0 (0.0) | 0 (0.0) | 0 (0.0) | 0 (0.0) | – |
| 31 to 180-day PPM/ICD implantation | 3 (0.7) | 2 (0.9) | 0 (0.0) | 0 (0.0) | 1 (3.1) | 0 (0.0) | 0 (0.0) | 0.36 |
| 31 to 180-day death | 6 (1.4) | 2 (0.9) | 0 (0.0) | 1 (1.6) | 1 (3.1) | 0 (0.0) | 2 (2.0) | 0.53 |
| 31 to 180-day PPM/ICD implantation or death | 9 (2.0) | 4 (1.9) | 0 (0.0) | 1 (1.6) | 2 (6.3) | 0 (0.0) | 2 (2.0) | 0.48 |

Values are n (%). No patient died within 30 days after TAVI procedure. CHB = complete heart block; HAVB = high-degree atrioventricular block; ICD = implantable cardioverter defibrillator; PPM = permanent pacemaker; TPM = temporary pacemaker.

**eTable 6. Pre-TAVI conduction disturbances and risk of HAVB/CHB and PPM/ICD implantation**

|  | **Total n** | **30-day HAVB/CHB,**  **n (%)** | **Procedural HAVB/CHB,**  **n (%)** | **Delayed HAVB/CHB,**  **n (%)** | **In-hospital**  **PPM/ICD**  **implantation,**  **n (%)** | **Discharge to 30-day PPM/ICD**  **implantation,**  **n (%)** |
| --- | --- | --- | --- | --- | --- | --- |
| **All patients** | **808** | 35 (4.3) | 23 (2.8) | 12 (1.5) | 24 (3.0) | 7 (0.9) |
| 1st-degree AVB | 181 | 12 (6.6) | 5 (2.8) | 7 (3.9) | 10 (5.5) | 1 (0.6) |
| RBBB | 115 | 18 (15.7) | 16 (13.9) | 1 (0.9) | 10 (8.7) | 3 (2.6) |
| LBBB | 45 | 1 (2.2) | 1 (2.2) | 0 (0.0) | 1 (2.2) | 1 (2.2) |
| IVCD | 38 | 1 (2.6) | 0 (0.0) | 1 (2.6) | 0 (0.0) | 0 (0.0) |

Values are n (%). AVB = atrioventricular block; CHB = complete heart block; HAVB = high-degree atrioventricular block; ICD = implantable cardioverter defibrillator; IVCD = interventricular conduction delay; LBBB = left bundle branch block; PPM = permanent pacemaker; RBBB = right bundle branch block; TAVI = transcatheter aortic valve implantation.

**eTable 7. Predictability of conduction disturbances for delayed HAVB/CHB among patients without procedural HAVB/CHB (n=806)**

|  | **Sensitivity** | **Specificity** | **Positive predictive value** | **Negative predictive value** |
| --- | --- | --- | --- | --- |
| **Pre-existing conduction disturbances** |  |  |  |  |
| 1st degree AVB | 58% | 78% | 3.9% | 99% |
| RBBB | 17% | 88% | 2.0% | 99% |
| LBBB | 0% | 94% | 0.0% | 98% |
| Wide QRS | 25% | 78% | 1.7% | 99% |
| **ECG findings at the end of procedure** |  |  |  |  |
| New-onset LBBB | 25% | 90% | 3.8% | 99% |
| ΔPR ≥20 ms increase | 8.33% | 65% | 0.4% | 98% |
| ΔQRS ≥20 ms increase | 42% | 84% | 3.8% | 99% |
| ΔPR or/and ΔQRS ≥20 ms increase | 50% | 55% | 1.6% | 99% |
| PR ≥240 ms | 42% | 87% | 4.7% | 99% |
| QRS ≥150 ms | 0% | 100% | Not calculable | 98% |
| PR ≥240 ms or/and QRS ≥150 ms | 42% | 87% | 4.7% | 99% |

AVB = atrioventricular block; CHB = complete heart block; ECG = electrocardiogram; HAVB = high-degree atrioventricular block; LBBB = left bundle branch block; RBBB = right bundle branch block; TAVI = transcatheter aortic valve implantation; TPM = temporary pacemaker.
